# Climbing strategies of Taiwan climbers

**DOI:** 10.1186/s40529-023-00399-4

**Published:** 2023-09-22

**Authors:** Po-Hao Chen, An-Ching Chung, Hung-Chih Lin, Sheng-Zehn Yang

**Affiliations:** 1 Liouguei Research Center, Taiwan Forest Research Institute, Liouguei District, Kaohsiung, Taiwan; 2 Graduate Institute of Bioresources, National Pingtung University of Science and Technology, No. 1, Shuefu Rd., Neipu 91201 Pingtung, Taiwan; 3 Division of Silviculture, Taiwan Forest Research Institute, No. 53, Nanhai Rd., 100051 Taipei, Taiwan; 4 Department of Forestry, National Pingtung University of Science and Technology, No. 1, Shuefu Rd., Neipu 91201 Pingtung, Taiwan

**Keywords:** Adventitious roots, Adhesive pads, Climbers, Hooks, Modified organs, Prehensile, Strategies, Speculation, Tendrils

## Abstract

**Background:**

The climbing strategies of lianas and herbaceous vines influence climber competition abilities and survival. The aim of this study was to investigate the climbing strategies of each plant species and observe their organs of origin.

**Results:**

The results showed that all Taiwan climbers were approximately 555 species, accounting for 11% of the native flora. Among the 555 climbers, the twining stem type was the most common, with a total of 255 species (46%), the remaining climbing methods accounted for 300 species. Approximately twenty one climbing methods, including nine combination types, were exhibited, of which the most common type was the twining stem, followed by simple scrambling and twining tendrils. Most species of Fabaceae and Apocynaceae were twining stems in dextrorse, excluding *Wisteriopsis reticulata* and *Alyxia taiwanensis*, which were in sinistrorse. The prehensile branch of *Fissi*s*tigma* genus, *Ventilago* genus, and *Dalbergia benthamii*, originated from second-order or modified stems. In the simple scrambling type, some climbers were covered spines and prickles to attach the host, and the others were clinging to the supports or creeping on the ground without speculation. The hooks or grapnels of the genus *Uncaria* are derived from the branches, and a pair of curved hooks or a spine of *Artabotrys hexapetalus* are originated from the inflorescence to tightly attach to a host. The *Piper* genus use adhesive roots to climb their hosts. Among the genus *Trichosanthes*, only *Trichosanthes homophylla* exhibits a combination of twining modified shoots and adhesive roots. Gentianales includes four families with seven climbing mechanisms, while Fabales includes only Fabaceae, which presents six climbing methods.

**Conclusions:**

The twining tendrils had nine organs of origin in Taiwan climber, that these opinions of originated organs might be available to the studies of convergent evolution. The data presented herein provide crucial basic information of the climber habits types and origin structures, which are available for terms standardization to improve field investigation. The terminologies would aid in the establishment of climber habits as commonly taxon-specific and the combination of two climber habits could be a characteristic of taxonomic value.

**Supplementary Information:**

The online version contains supplementary material available at 10.1186/s40529-023-00399-4.

## Background

Climbing plants germinate on the ground and develop for a certain period; their stems need external support to sustain themselves mechanically. Climbing plants or climbers were used to describe plants displaying climbing habits, and lianas and vines were used to describe woody and herbaceous climbers, respectively (Sperotto et al. 2020). After establishing themselves on hosts, some climbers still connect to the ground, while others begin to lose their function from the tip of the stem and lose their connection to the ground (Moffett 2000). The latter ultimately loses contact with the soil and becomes epiphytic, which is named the nomadic vine, nomadic climber, or secondary hemiepiphytes (Moffett 2000).

Climbing plants use different climbing strategies to develop and establish their abundance and survival. The stems of climbers have a twining function, which is the most crucial climbing method for climbing plants (Muthuramkumar and Parthasarathy 2000). Twining stems are divided into two subtypes: dextrorse, which is a left-to-right spiral when viewed from the front, and sinistrorse, which is a right-to-left spiral when viewed from the front (Edwards et al. 2007; Beentje 2010; Burnham and Revilla-Minaya 2011; Wang et al. 2013). Twining tendrils are terminal, haptotropic, thread-like structures that are used exclusively for climbing (Darwin 1865; Sousa-Baena et al. 2018). Darwin (1865) proposed that tendrils are filamentous structures that wrap around other objects via helical growth. Therefore, a special structure with a filamentous shape is generally referred to as twining tendrils, and the tendrils of the family Cucurbitaceae developed in the leaf axils are theorized to represent modified flowers (Darwin 1865), leaves (Sensarma 1955), and shoots or second-order branches (Sensarma 1955; Gerrath et al. 2008).

The thorns or other spines covering the climbers serve a defensive function in addition to helping the hosts climb. For example, the *Mimosa* genus (Fabaceae) includes several thorny species, not only for climbing but also for protection against predators (Barneby 1991). In shaded regions or the understory of forests, younger plants increase thorn production in *Artabotrys hexapetalus* to avoid being bitten and increase their climbing abilities (Fisher et al. 2002).

Adventitious roots are a climbing type that can adsorb onto trees and rock walls. The adhesive roots of climbers secrete polysaccharides and proteins from the root hairs on the adventitious roots, and the adventitious roots and root hairs attach to produce an adhesive pad that can be adsorbed on any substrate (Groot et al. 2003). Areas with shorter dry seasons and higher average annual precipitation have more adhesive root species (Durigon et al. 2013).

Taiwan is located in the subtropical monsoon region. The climate is warm and humid throughout the year, with temperatures of approximately 22–24 °C and annual average precipitation of approximately 2000–2500 mm. Owing to their favorable environment, diverse and abundant species are present, including climbing plants, with approximately 52 families and 287 liana species distributed in Taiwan (Yang et al. 2022). The climbing strategies of climbers in central and southern Taiwan have been previously investigated (Chen et al. 2013). In this study, we continued to study the climbing methods of all Taiwan climbers. We hope that diverse climbing modes will become taxonomic features that contribute to plant classification and will ultimately be integrated into conservation research on global climbing plant diversity.

## Materials and methods

In this study, we concentrate solely on flowering plants of Taiwan climbers, although some plants of ferns and lycophytes, Orchidaceae, and Poaceae, conform the definition of lianas. The categorized families table will be arranged according to the APG IV system (Stevens 2001). The climbing strategies we used were based on Sperotto et al. (2020) and were divided into active and passive climbing types. Active climbing types were divided into the following: (1) Twining stem, where the climber stem has a twining function and has two groups: dextrorse and sinistrorse. (2) Twining tendrils, where the tendril is defined as branches, leaves, stipules, and inflorescences specializing in tendril twining around support without stem twining, thin, short, wrapping, or grasping structures with hooks or adhesive pads at the ends. (3) Twining leaf petioles, where petioles were twined around the support. (4) Prehensile branch, where the lateral leaf-bearing branches have a twining function. This is different from using stems, such as tendrils, which function and twine around the support; they do not possess any type of structural modification but originate from shoot-modified or second-order branches. (5) Twining peduncles or inflorescences, where the peduncles or inflorescences are modified to twine with the host.

The passive climbing types were classified as (1) simple scrambling, whereas climbers may or may not have spines, prickles, and thorns to support, and without hooks or grapnels. (2) Hooks or grapnels that plants bear hooks or grapnels to scramble the host; these are specialized structures. (3) Adhesive roots, where the adventitious roots of climbers can be adsorbed onto trees and rock walls.

The origins of the tendrils were divided into two categories with 17 types, as described by Sousa-Baena et al. (2018). The first tendril category originates from ten types of vegetative organs, and the second tendril category originated from seven types of reproductive organs. The first category includes the following: (1) modified terminal leaflets; (2) prolonged midrib; (3) prolonged forked tips of the midribs; (4) modified petioles and a transitory structure that develops other functions, except climbing, in later developmental stages of leaves; (5) modified leaf tip; (6) whole leaf modified into a simple tendril; (7) petiole duplication; (8) modified petioles that develops twining capacity; (9) modified compound leaf rachis that acquires the capacity for helical growth, becoming voluble; and (10) a modified shoot. The second category includes (1) tip of the reduced inflorescence apex; (2) modified whole inflorescences; (3) modified inflorescence apices; (4) modified inflorescence rachis that acquires the capacity for helical growth, becoming voluble; (5) inflorescence lateral branches; (6) inflorescence peduncles; and (7) flower pedicels that acquire the capacity for helical growth.

The scientific names of the climbers were determined according to the Flora of Taiwan 2nd ed. (Huang et al. 1993–2003). We referred to some taxonomic revisions of the families Araceae (Croat 1981), Aristolochiaceae (Zhu et al. 2019), Asclepiadoideae (Hsu et al. 2021), The Red List of Vascular Plants of Taiwan (Editorial Committee of the Red List of Taiwan Plant 2017), Convolvulaceae (Simões and Staples 2017; Chao et al. 2019), Fabaceae (Pan and Zhu 2010; Maslin et al. 2013; Compton et al. 2019; Song and Pan 2022), Opiliaceae (Chen et al. 2020), *Macrotyloma axillare* (Chen et al. 2021), Passifloraceae (Chen et al. 2022), Piperaceae (Chang and Kung 2020), Rosaceae (Huang and Hu 2009), Rubiaceae (Razafimandimbison & Bremer 2011), Schisandraceae (Suetsugu et al. 2017), and Vitaceae (Wen et al. 2014, 2018; Parmar et al. 2021). Each climbing mechanism was arranged by family name in alphabetical order. All the collected specimens were deposited in the herbarium of Provincial Pingtung Institute (PPI) at the National Pingtung University of Science and Technology, Pingtung, Taiwan, for subsequent identification.

## Results and discussion

Among the 555 Taiwan climbers (Table 1, Additional file 1: Appendix S1), the twining stem type was the most common, with a total of 255 species (46%), including 217 species that were dextrorse and 38 species that were sinistrorse. The remaining climbing methods accounted for 300 species (54%), including 104 species of simple scrambling, 59 species of twining tendrils (33 species of twining modified shoot, 15 species of twining petiole duplication, 10 species of twining terminal leaflets, and 1 species of twining leaf tip), 36 species of adhesive roots, 36 species of twining peduncles or inflorescence, 23 species of twining petioles, seven species of prehensile branch, five species of hooks or grapnels scrambling. The 30 climbers used a combination of two or three climbing mechanisms and a total of ten combination types. Approximately 19 species belonged to the simple scrambling type but were without spines, prickles, or thorns. Most families had only one climbing method; among them, Fabaceae (85 spp.) had the highest number of climbing methods (six). Rubiaceae had five climbing methods but only eighteen species. We then described each climbing mechanism in Taiwan climbers by active and passive climbing types and observed tendril-origin vegetative and reproductive organs.

**Table 1:** Climbing methods of different climber families in Taiwan

Family	TSd	TSs	TMS	TPD	TTL	TLt	TLp	TPI	PB	SS	AR	HG	TPD + SS	TSd + TSs	TSd + AR	TSs + SS	TPI + AR	TSd + SS	TMS + AR	HG + SS	TSd + TSs + SS	SP no	CM no
1 Acanthaceae	5	–	–	–	–	–	–	–	–	–	–	–	–	–	–	–	–	–	–	–	–	5	1
2 Actinidiaceae	5	–	–	–	–	–	–	–	–	–	–	–	–	–	–	–	–	–	–	–	–	5	1
3 Amaranthaceae	–	–	–	–	–	–	–	–	–	1	–	–	–	–	–	–	–	–	–	–	–	1	1
4 Anacardiaceae	–	–	–	–	–	–	–	–	–	–	1	–	–	–	–	–	–	–	–	–	–	1	1
5 Annonaceae	–	–	–	–	–	–	–	–	2	–	–	–	–	–	–	–	–	–	–	1	–	3	2
6 Apocynaceae	27	1	–	–	–	–	–	–	–	–	1	–	–	–	4	–	–	–	–	–	–	33	4
7 Araceae	–	–	–	–	–	–	–	–	–	–	8	–	–	–	–	–	–	–	–	–	–	8	1
8 Araliaceae	–	–	–	–	–	–	–	–	–	2	1	–	–	–	–	–	–	–	–	–	–	3	2
9 Arecaceae	–	–	–	–	–	–	–	–	–	–	–	2	–	–	–	–	–	–	–	–	–	2	1
10 Aristolochiaceae	5	–	–	–	–	–	–	–	–	–	–	–	–	–	–	–	–	–	–	–	–	5	1
11 Asparagaceae	–	–	–	–	–	–	–	–	–	–	–	–	–	–	–	1	–	–	–	–	–	1	1
12 Asteraceae	–	–	–	–	–	–	–	–	–	5	–	–	–	3	–	–	–	–	–	–	–	8	2
13 Basellaceae	2	–	–	–	–	–	–	–	–	–	–	–	–	–	–	–	–	–	–	–	–	2	1
14 Bignoniaceae	–	–	–	–	2	–	–	–	–	–	–	–	–	–	–	–	–	–	–	–	–	2	1
15 Campanulaceae	1	–	–	–	–	–	–	–	–	–	–	–	–	1	–	–	–	–	–	–	–	2	2
16 Cannabaceae	–	1	–	–	–	–	–	–	–	–	–	–	–	–	–	–	–	–	–	–	–	1	1
17 Capparaceae	–	–	–	–	–	–	–	–	–	5	–	–	–	–	–	–	–	–	–	–	–	5	1
18 Caprifoliaceae	–	5	–	–	–	–	–	–	–	–	–	–	–	–	–	–	–	–	–	–	–	5	1
19 Celastraceae	5	–	–	–	–	–	–	–	–	–	2	–	–	–	–	–	–	–	–	–	–	7	2
20 Combretaceae	–	–	–	–	–	–	–	–	–	–	–	–	–	–	–	–	–	1	–	–	–	1	1
21 Connaraceae	–	–	–	–	–	–	–	–	2	–	–	–	–	–	–	–	–	–	–	–	–	2	1
22 Convolvulaceae	54	–	–	–	–	–	–	–	–	4	–	–	–	–	–	–	–	–	–	–	–	58	2
23 Cucurbitaceae	–	–	31	–	–	–	–	–	–	–	–	–	–	–	–	–	–	–	1	–	–	32	2
24 Dioscoreaceae	6	4	–	–	–	–	–	–	–	–	–	–	–	–	–	3	–	1	–	–	–	14	4
25 Elaeagnaceae	–	–	–	–	–	–	–	–	–	7	–	–	–	–	–	–	–	–	–	–	–	7	1
26 Euphorbiaceae	–	–	–	–	–	–	–	–	–	1	–	–	–	–	–	–	–	–	–	–	–	1	1
27 Fabaceae	68	1	1	–	8	–	–	–	1	6	–	–	–	–	–	–	–	–	–	–	–	85	6
28 Flagellariaceae	–	–	–	–	–	1	–	–	–	–	–	–	–	–	–	–	–	–	–	–	–	1	1
29 Gentianaceae	–	7	–	–	–	–	–	–	–	2	–	–	–	–	–	–	–	–	–	–	–	9	2
30 Gesneriaceae	–	–	–	–	–	–	–	–	–	–	1	–	–	–	–	–	–	–	–	–	–	1	1
31 Heliotropiaceae	–	–	–	–	–	–	–	–	–	1	–	–	–	–	–	–	–	–	–	–	–	1	1
32 Hernandiaceae	–	–	–	–	–	–	1	–	–	–	–	–	–	–	–	–	–	–	–	–	–	1	1
33 Hydrangeaceae	–	–	–	–	–	–	–	–	–	–	4	–	–	–	–	–	–	–	–	–	–	4	1
34 Lamiaceae	–	–	–	–	–	–	–	–	–	1	–	–	–	1	–	–	–	–	–	–	–	2	2
35 Lardizabalaceae	5	–	–	–	–	–	–	–	–	–	–	–	–	–	–	–	–	–	–	–	–	5	1
36 Lauraceae	2	–	–	–	–	–	–	–	–	–	–	–	–	–	–	–	–	–	–	–	–	2	1
37 Loganiaceae	2	–	1	–	–	–	–	–	–	–	–	–	–	–	–	–	–	–	–	–	–	3	2
38 Malpighiaceae	3	–	–	–	–	–	–	–	–	–	–	–	–	–	–	–	–	–	–	–	–	3	1
39 Malvaceae	–	–	–	–	–	–	–	–	–	1	–	–	–	–	–	–	–	–	–	–	–	1	1
40 Melastomataceae	–	–	–	–	–	–	–	–	–	2	–	–	–	–	–	–	–	–	–	–	–	2	1
41 Menispermaceae	14	–	–	–	–	–	–	–	–	–	–	–	–	–	–	–	–	–	–	–	–	14	1
42 Moraceae	1	–	–	–	–	–	–	–	–	1	7	–	–	–	–	–	–	–	–	–	–	9	3
43 Nyctaginaceae	–	–	–	–	–	–	–	–	–	2	–	–	–	–	–	–	–	–	–	–	–	2	1
44 Oleaceae	4	–	–	–	–	–	–	–	–	–	–	–	–	–	–	–	–	–	–	–	–	4	1
45 Opiliaceae	–	–	–	–	–	–	–	–	–	1	–	–	–	–	–	–	–	–	–	–	–	1	1
46 Pandanaceae	–	–	–	–	–	–	–	–	–	–	1	–	–	–	–	–	–	–	–	–	–	1	1
47 Passifloraceae	–	–	–	–	–	–	–	8	–	–	–	–	–	–	–	–	–	–	–	–	–	8	1
48 Phyllanthaceae	–	–	–	–	–	–	–	–	–	1	–	–	–	–	–	–	–	–	–	–	–	1	1
49 Piperaceae	–	–	–	–	–	–	–	–	–	1	8	–	–	–	–	–	–	–	–	–	–	9	2
50 Polygonaceae	–	–	–	–	–	–	–	1	–	3	–	–	–	1	–	–	–	–	–	–	–	5	3
51 Primulaceae	1	1	–	–	–	–	–	–	–	–	–	–	–	–	–	–	–	–	–	–	1	3	3
52 Ranunculaceae	–	–	–	–	–	–	21	–	–	–	–	–	–	–	–	–	–	–	–	–	–	21	1
53 Rhamnaceae	–	5	–	–	–	–	–	–	2	3	–	–	–	–	–	–	–	–	–	–	–	10	3
54 Rosaceae	–	–	–	–	–	–	–	–	–	46	–	–	–	–	–	–	–	–	–	–	–	46	1
55 Rubiaceae	4	6	–	–	–	–	–	–	–	4	1	3	–	–	–	–	–	–	–	–	–	18	5
56 Rutaceae	–	–	–	–	–	–	–	–	–	3	–	–	–	–	–	–	–	–	–	–	–	3	1
57 Sabiaceae	2	–	–	–	–	–	–	–	–	–	–	–	–	–	–	–	–	–	–	–	–	2	1
58 Sapindaceae	–	–	–	–	–	–	–	1	–	–	–	–	–	–	–	–	–	–	–	–	–	1	1
59 Schisandraceae	–	5	–	–	–	–	–	–	–	–	–	–	–	–	–	–	–	–	–	–	–	5	1
60 Smilacaceae	–	–		15	–	–	–	–	–	–	–	–	8	–	–	–	–	–	–	–	–	23	2
61 Solanaceae	–	2	–	–	–	–	1	–	–	–	–	–	–	–	–	–	–	–	–	–	–	3	2
62 Stachyuraceae	–	–	–	–	–	–	–	–	–	1	–	–	–	–	–	–	–	–	–	–	–	1	1
63 Stemonaceae	1	–	–	–	–	–	–	–	–	–	–	–	–	–	–	–	–	–	–	–	–	1	1
64 Urticaceae	–	–	–	–	–	–	–	–	–	–	1	–	–	–	–	–	–	–	–	–	–	1	1
65 Vitaceae	–	–			–	–	–	26	–	–	–	–	–	–	–	–	3	–	–	–	–	29	3
Σ Families	21	12	3	1	2	1	3	4	4	23	12	2	1	4	1	2	1	2	1	1	1		
Σ Species	217	38	33	15	10	1	23	36	7	104	36	5	8	6	4	4	3	2	1	1	1	555	
%	39.1	6.9	6.0	2.7	1.8	0.2	4.1	6.5	1.3	18.7	6.5	0.9	1.4	1.1	0.7	0.7	0.5	0.4	0.2	0.2	0.2	100	

*TS* twining stem, *TSd* twining stem in dextrorse, *TSs* twining stem in sinistrorse, *TMS* twining modified shoot, *TTL* twining terminal leaflets, *TLt* twining leaf tip, *TPD* twining petiole duplication, *TLp* twining leaf petioles, *TPI* twining peduncles or inflorescence, *PB* prehensile branch (also called TB: twining lateral branch), *SS* simple scrambling, *AR* adhesive roots, or adhesive pads, *HG* hooks or grapnels, *SP no.* species number, *CM no.* climbing methods number. *TT* twining tendrils (including TMS, TTL, TLt, TPD)

### Active climbing types

#### 1. Twining stem

Approximately 13 families have unique twining stems (dextral), namely Acanthaceae, Actinidiaceae, Aristolochiaceae, Basellaceae, Combretaceae, Convolvulaceae, Lardizabalaceae, Lauraceae, Malpighiaceae, Menispermaceae, Oleaceae, Sabiaceae, and Stemonaceae (Table 1); and four families have twining stem in sinistrorse, namely Cannabaceae, Caprifoliaceae, Gentianaceae, and Schisandraceae. Three families have twining stems (both dextral and sinistral): Asteraceae, Campanulaceae, and Dioscoreaceae. Approximately the same proportions of twining stems are present in dextrorse and sinistrorse in Dioscoreaceae.

**Fig. 1:**
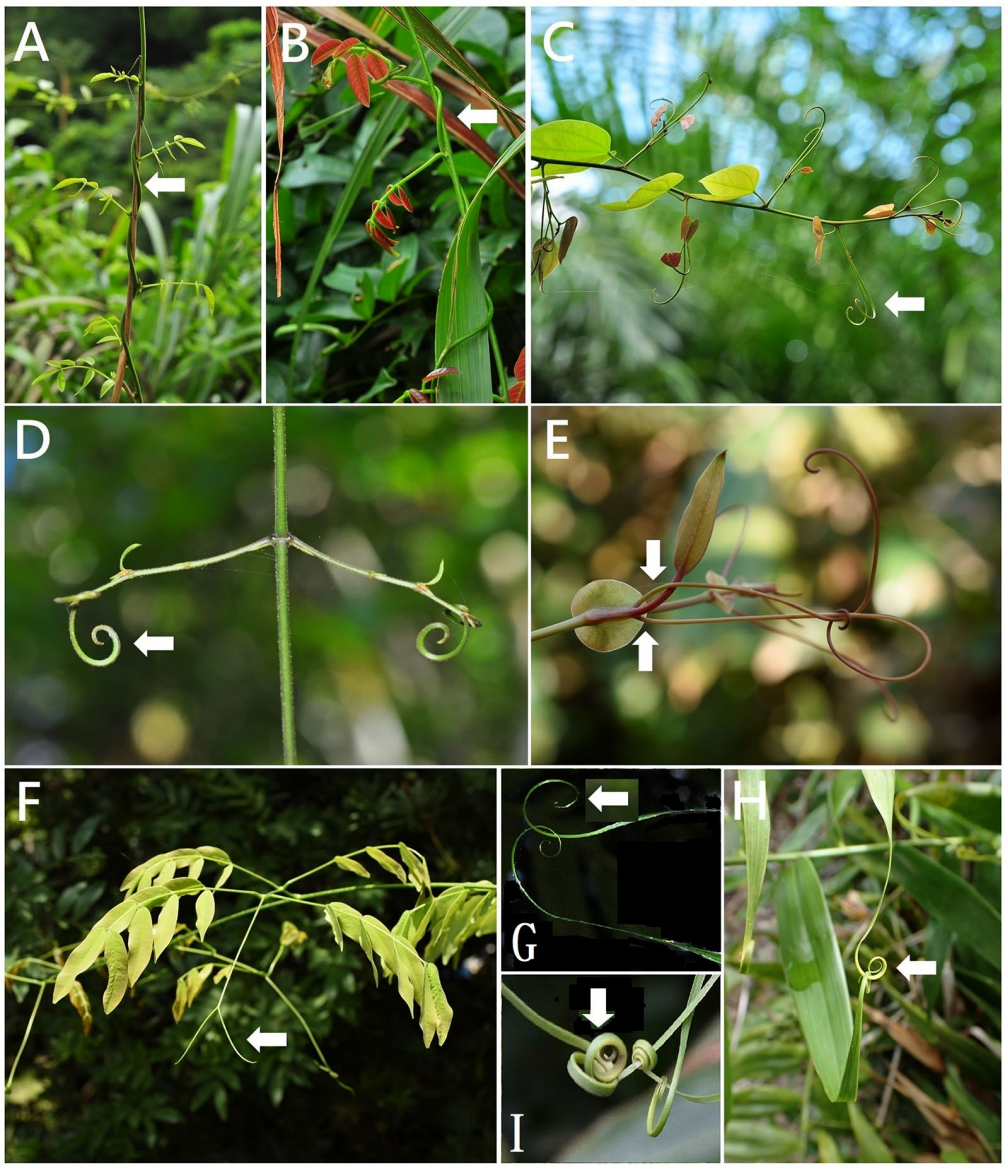
**A** *Wisteriopsis reticulata* (Fabaceae): twining stems in sinistrorse. **B** *Derris laxiflora* (Fabaceae): twining stems in dextrorse. **C** *Phanera championii* (Fabaceae): tendrils derived from a modified shoot and lignified. **D** *Strychnos cathayensis* (Loganiaceae): tendrils derived from a modified shoot and lignified. **E** *Smilax ocreata* (Smilacaceae): tendrils derived from petiole duplication. **F** *Entada rheedei* (Fabaceae): tendrils derived from modified terminal leaflets. **G** *Flagellaria indica* (Flagellariaceae): tendrils derived from a prolonged leaf tip. **H** *F. indica*: two prolonged leaf tips twining. **I** *F. indica*: tendrils enlarged

In the Fabaceae family, 68 species had twining stems in dextrorse, and only one species, *Wisteriopsis reticulata*, is in sinistrorse (Fig. 1A). The leaf morphologies of *Derris laxiflora* (Fig. 1B) and *W. reticulata* were similar and difficult to distinguish, and the climber mechanisms of dextrorse and sinistrorse were available for the identification of these two species. These two mechanisms are also the basis for distinguishing *Wisteria sinensis* from *Wisteria floribunda*; the former is dextrorse, and the latter is sinistrorse (Wang et al. 2013). Similarly, 35 species in Apocynaceae are twining stems in dextrorse; only *Alyxia taiwanensis* is twining stems in sinistrorse; indeed, this sinistrorse type is a diagnostic feature of this species.

#### 2. Twining tendrils

The twining tendrils type was exhibited in Bignoniaceae, Cucurbitaceae, Flagellariaceae, Passifloraceae, Sapindaceae, Smilacaceae, Vitaceae. According to the definition in Sousa-Baena et al. (2018) and Wu et al. (1994–2004), five tendril modes originating from vegetative organs were divided (Table 2).

- 1. Modified shoots

**Table 2:** Organs of origin of tendrils in Taiwan climbers

Orders	Family	Species	Organs of origin by Sousa–Baena et al. (2018) and Wu et al. (1994–2004)
Caryophyllales	Polygonaceae	*Antigonon leptopus*	Modified inflorescence apices, tendrils 2–3
Malpighiales	Passifloraceae	*Passiflora*	Tip of the reduced inflorescence apex
Sapindales	Sapindaceae	*Cardiospermum halicacabum*	tendril pairs at the modified inflorescence rachis base
Vitales	Vitaceae	*Ampelopsis*	Whole modified inflorescence into tendrils
..	..	*Nekemias cantoniensis*	..
..	..	*Vitis*	..
Vitales	Vitaceae	*Cissus*	Whole modified inflorescence /modified extra-axillary branch, adhesive pads +/−
..	..	*Parthenocissus tricuspidata*	..
..	..	*Tetrastigma*	..
Cucurbitales	Cucurbitaceae	*Citrullus, Coccinia*	A modified shoot, tendrils lignified
..	..	*Cucurbita, Cucumis*	..
..	..	*Momordica, Mukia*	..
Fabales	Fabaceae	*Phanera championii*	..
Gentianales	Loganiaceae	*Strychnos cathayensis*	..
Laurales	Hernandiaceae	*Illigera luzonensis*	Petioles that acquire the capacity for helical growth
Laurales	Menispermaceae	*Cissampelos*	..
Ranunculales	Ranunculaceae	*Clematis*	..
Solanales	Solanaceae	*Solanum seaforthianum*	..
Fabales	Fabaceae	*Entada*	Modified terminal leaflet/determined rachis
..	..	*Vicia*	..
Lamiales	Bignoniaceae	*Pyrostegia venusta*	..
Poales	Flagellariaceae	*Flagellaria indica*	Prolonged leaf tip/thickened prolonged leaf midrib
Solanales	Smilacaceae	*Smilax*	Modified stipule, petiole duplication, a pair of tendrils
Cucurbitales	Cucurbitaceae	*Thladiantha*	Shoot-stipule complex
Cucurbitales	Cucurbitaceae	*Neoalsomitra*	Modified shoot, adhesive pads +/−
..	..	*Trichosanthes homophylla*	..

From ontogenetic studies on shoot-derived tendrils conducted on Cucurbitaceae (Sousa-Baena et al. 2018), we documented that the tendrils of *Citrullus*,* Coccinia*,* Cucumis*,* Cucurbita*,* Momordica*, *Mukia*, *Phanera championii* (Fabaceae) (Fig. 1C)*,* and *Strychnos cathayensis* (Loganiaceae) (Fig. 1D) (Table 2) were from modified young shoots, stems, or second-order branches and gradually lignified.

- 2. Terminal leaflets

The tendrils of three *Entada* species (Fabaceae) (Fig. 1F), five *Vicia* species (Fabaceae), and *Pyrostegia venusta* (Bignoniaceae) originated from terminal leaflets.

- 3. Prolonged leaf tip

*Flagellaria indica* exhibited a prolonged leaf tip or thickened leaf midrib (Fig. 1G–I).

- 4. Petiole duplication

A pair of tendrils of the Taiwan *Smilax* species (Fig. 1E) originating from petiole duplication is consistent with previous reports.

- 5. Modified shoots and adhesive roots

The tendrils of *Thladiantha* genus were complexes produced by shoots and stipules, and two genera, *Neoalsomitra* and *Trichosanthes*, had a combination of modified shoots and adhesive roots (Table 2); however, these characteristics must still be investigated.

#### 3. Twining petioles

**Fig. 2:**
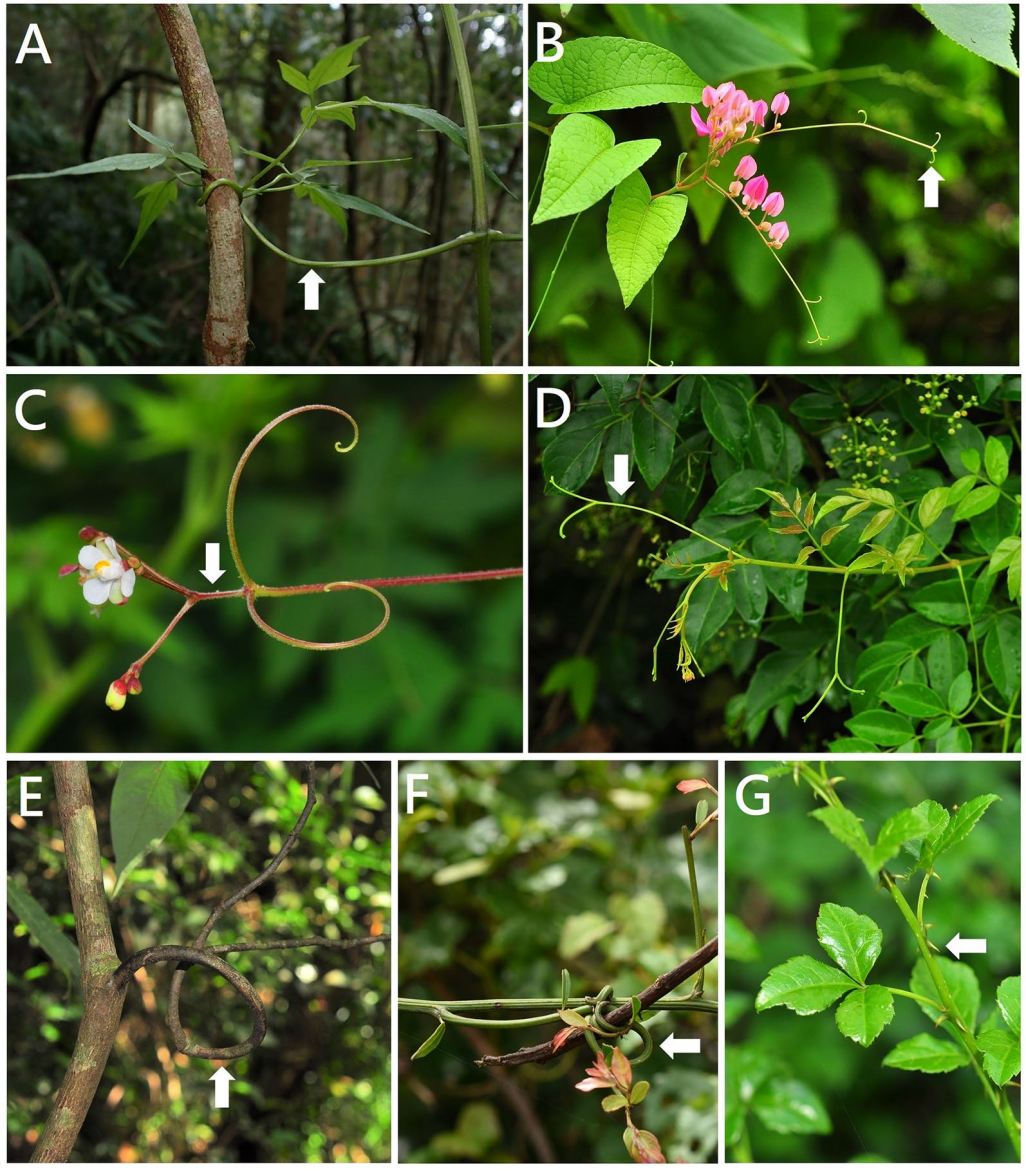
**A** *Clematis tamurae* (Ranunculaceae): tendrils derived from twining leaf petioles*.* **B** *Antigonon leptopus* (Polygonaceae): tendrils derived from modified inflorescence apices. **C** *Cardiospermum halicacabum* (Sapindaceae): tendrils derived inflorescence rachis and a pair of tendrils at the rachis base*.* **D** *Nekemias cantoniensis* (Vitaceae): tendrils derived from whole inflorescence modified into tendrils. **E** *Fissistigma glaucescens* (Annonaceae): prehensile branches. **F** *Ventilago elegans* (Rhamnaceae): prehensile branches. **G** *Eleutherococcus trifoliatus* (Araliaceae): simple scrambling

Approximately 21 *Clematis* species (Ranunculaceae) (Fig. 2A), *Illigera luzonensis* (Hernandiaceae), and *Solanum seaforthianum* (Solanaceae) had unique climbing methods, namely twining petioles. We observed a twining stem (dextrorse) for *Cissampelos pareira* var. *hirsuta* (Menispermaceae), which was inconsistent with the twining petiole of the genus *Cissampelos* (Table 2) (Sousa-Baena et al. 2018), and further investigation is required.

#### 4. Twining peduncles or inflorescence

There are four tendril modes originating from reproductive organs in this study (Table 2). *Antigonon leptopus* (Polygonaceae) had twining tendrils of the inflorescence apex, and its tendrils originated from a modified inflorescence apex and formed two to three tendrils (Fig. 2B). *Cardiospermum halicacabum* (Sapindaceae) had a twining tendril of the modified inflorescence rachis that formed a pair of tendrils at the rachis base (Fig. 2C). The peduncles of *Passiflora* genus (Passifloraceae) were often degenerated or absent, the central axis developed into a tendril, and the secondary axes were reduced to one to two flowers (Wu et al. 1994–2004). The twining tendrils of the eight Taiwanese *Passiflora* species originated from the tip of the reduced inflorescence apex. In the Vitaceae family, the inflorescence rachis of *Ampelopsis*,* Nekemias cantoniensis* (Fig. 2D), and *Vitis* genus had the helical growth capacity to climb hosts and were named modified inflorescence tendrils. The genera *Cissus*,* Tetrastigma*, and *Parthenocissus tricuspidata* modified the inflorescent tendrils combined with adhesive pads (Sousa-Baena et al. 2018). In this study, we did not find any *Cissus* genus with this combination type; therefore, this needs to be investigated.

#### 5. Prehensile branch

Prehensile branches were found in Annonaceae, Connaraceae, Fabaceae, and Rhamnaceae. The lateral leaf-bearing branches had a twining function different from that of using the stem; this climbing mechanism was named the prehensile branch or twining lateral branch. In this study, approximately seven species (Table 1) had a prehensile branch, including *Fissistigma glaucescens*,* F. oldhamii* (Annonaceae) (Fig. 2E)*, Connarus subinaequifolius* (Connaraceae), *Rourea minor* (Connaraceae), *Dalbergia benthamii* (Fabaceae), and *Ventilago elegans*,* V. leiocarpa* (Rhamnaceae) (Fig. 2F). The species *C. subinaequifolius* was only distributed in Lanyu, and few individuals were found, which influenced the observation of the climbing method. According to Sperotto et al. (2020), we classified it as a twining lateral branch (prehensile branch).

### Passive climbing types

#### 1. Simple scrambling

**Fig. 3:**
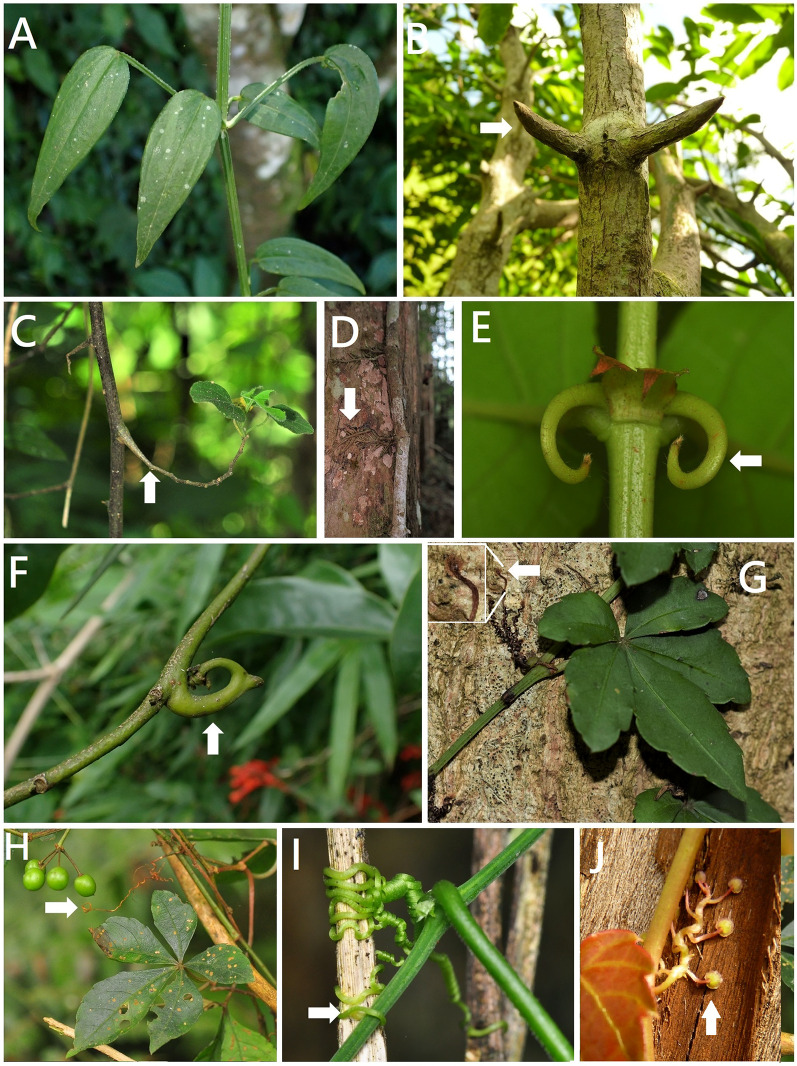
**A** *Rubia lanceolata* (Rubiaceae): simple scrambling. **B** *Artabotrys hexapetalus* (Annonaceae): simple scrambling. **C** *Mallotus repandus* (Euphorbiaceae): simple scrambling. **D** *Piper kadsura* (Piperaceae): adhesive roots*.* **E**. *Uncaria lanosa* var. *appendiculata* (Rubiaceae): hooks or grapnels. **F** *Artabotrys hexapetalus* (Annonaceae): hooks or grapnels. **G**, **H** *Tetrastigma obtectum* var. *glabrum* (Vitaceae): tendrils derived from modified inflorescence with terminal adhesive roots. **I** *Trichosanthes homophylla* (Cucurbitaceae): tendrils derived from modified shoot with adhesive roots. **J** *Parthenocissus tricuspidata* (Vitaceae): tendrils derived from modified inflorescence with adhesive roots

Approximately 10 families bore simple scrambling, namely Asparagaceae, Capparaceae, Elaeagnaceae, Euphorbiaceae, Malvaceae, Nyctaginaceae, Opiliaceae, Phyllanthaceae, Rosaceae, Rutaceae, and included 104 climbers (18.7%). Among the 104 climbers (Tables 1 and 3), Rutaceae, Rosaceae, *Caesalpinia* genus,* Persicaria* genus,* Asparagus cochinchinensis*,* Eleutherococcus trifoliatus* (Fig. 2G),* Hibiscus surattensis*,* Mimosa diplotricha*, and *Senegalia caesia* developed prickles that were derived from the epidermis of the stems and lateral branches and were detachable without tearing the organ. The prickles of *E. trifoliatus, H. surattensis,* and *S. caesia* were either curved or recurved. In the family Smilacaceae, *Smilax arisanensis*,* S. bracteata *var*. bracteata*,* S. bracteata *var*. verruculosa*,* S. china*,* S. elongato-umbellata*,* S. horridiramula*,* S. ocreata*, and *S. sieboldii* had prickles covering the stem, and in the family Dioscoreaceae, *Dioscorea collettii*, *D. cumingii*,* D. esculenta* var. *spinosa*, and *D. matsudae* had prickles at their petiole base (Liao 2000). The simple scrambling combined with twining petiole duplication tendrils in Smilaceae and twining stems in Dioscoreaceae have become the basic diagnostic characteristics that aid twining around the host. The indumentum of the *Rubia* genus (Fig. 3A) was hair with raphides scrambling other plants. The nodes of *Artabotrys hexapetalus* (Annonaceae) (Fig. 3B) had paired plagiotropic branches, and occasionally each node had only a single plagiotropic branch. *Mallotus repandus* (Euphorbiaceae) (Fig. 3C) had spine lignified, straight, persistent with a sharp-pointed, hardened structure and derived from branches.

**Table 3:** Organs of origin of spiculates in Taiwan climbers

Order	Family	Species	Organs of origin by Beentje (2010), Wu et al. (1994–2004)
Apiales	Araliaceae	*Eleutherococcus trifoliatus*	Prickles derived from stems, lateral branches or leaves, prickles have a sharp outgrowth from the epidermis, detachable without tearing the organ
Asparagales	Asparagaceae	*Asparagus cochinchinensis*	..
Caryophyllales	Polygonaceae	*Persicaria*	..
Dioscoreales	Dioscoreaceae	*Dioscorea collettii, D. cumingii, D. esculenta* var. *spinosa, D. matsudae*	..
Fabales	Fabaceae	*Caesalpinia, Mimosa diplotricha*,* Senegalia caesia*	..
Liliales	Smilacaceae	*Smilax arisanensis, S. bracteata* var. *bracteata, S. bracteata* var. *verruculosa, S. china, S. elongato-umbellata, S. horridiramula, S. ocreata, S. sieboldii*	..
Malvales	Malvaceae	*Hibiscus surattensis*	..
Rosales	Rosaceae		..
Sapindales	Rutaceae		..
Brassicales	Capparaceae	*Capparis*	Spine lignified, straight, persistent with a sharp-pointed, hardened structure and derived from leaves, stipules, branches or petioles
Caryophyllales	Nyctaginaceae		..
Ericales	Primulaceae	*Embelia laeta* var.* papilligera*	..
Gentianales	Rubiaceae	*Randia sinensis*	..
Magnoliales	Annonaceae	*Artabotrys hexapetalus*	..
Malpighiales	Euphorbiaceae	*Mallotus repandus*	..
..	Phyllanthaceae	*Phyllanthus reticulatus*	..
Myrtales	Combretaceae	*Quisqualis indica*	..
Rosales	Rhamnaceae	*Rhamnus formosana*	..
..	Elaeagnaceae	*Elaeagnus*	..
..	Moraceae	*Maclura cochinchinensis*	..
..	Rhamnaceae	*Sageretia randaiensis*	..
Santalales	Opiliaceae	*Cansjera rheedei*	..
Gentianales	Rubiaceae	*Rubia*	Prickly and/or longitudinally ribbed or winged, hairs raphide present

The simple scrambling type indicates that a climber is with or without spines, prickles, or thorns (Sperotto et al. 2020). In this study, approximately 19 climbers did not have spiculates but could cling to the host or creep on the ground (Table 4). The species that clung to support included *Blumea riparia* var. *megacephala* (Asteraceae),* Deeringia amaranthoides* (Amaranthaceae), *Heliotropium sarmentosum* (Heliotropiaceae), *Medinilla formosana* (Melastomataceae), *Medinilla hayataina* (Melastomataceae), *Microglossa pyrifolia* (Asteraceae)*, Persicaria chinense* (Polygonaceae), *Senecio scandens* var. *scandens* (Asteraceae), *Vernonia elliptica* (Asteraceae)*,* and *Wedelia biflora* (Asteraceae). *Stachyurus himalaicus* (Stachyuraceae) had a shrub phase but could cling to support when it gradually increased in height. Four Convolvulaceae species, *Dichondra micrantha*, *Evolvulus nummularius*, *Ipomoea imperati*,* Ipomoea pes-caprae* subsp. *brasiliensis*, and *Piper sarmentosum* (Piperaceae), *Rubus pentalobus* (Rosaceae), *Tripterospermum cordifolium* (Gentianaceae), *Tripterospermum microphyllum* (Gentianaceae), and *Vitex rotundifolia* (Lamiaceae) exhibited creeping on the ground (Table 4).

**Table 4:** Climbers classified as simple scrambling without spiculates

Order	Family	Species	Climbing strategies
Asterales	Asteraceae	*Blumea riparia* var.* megacephala* *Microglossa pyrifolia* *Senecio scandens* var.* scandens* *Vernonia elliptica* *Wedelia biflora*	Clinging
Boraginales	Heliotropiaceae	*Heliotropium sarmentosum*	Clinging
Caryophyllales	Amaranthaceae	*Deeringia amaranthoides*	Clinging
..	Polygonaceae	*Persicaria chinense*	Clinging
Crossosomatales	Stachyuraceae	*Stachyurus himalaicu*s	Clinging
Myrtales	Melastomataceae	*Medinilla formosana*	Clinging
..	..	*Medinilla hayataina*	Clinging
Gentianales	Gentianaceae	*Tripterospermum cordifolium*	Creeping
..	..	*Tripterospermum microphyllum*	Creeping
Lamiales	Lamiaceae	*Vitex rotundifolia*	Creeping
Piperales	Piperaceae	*Piper sarmentosum*	Creeping
Solanales	Convolvulaceae	*Dichondra repens* *Evolvulus nummularius* *Ipomoea imperati* *Ipomoea pes-caprae* subsp.* brasiliensis*	Creeping

#### 2. Adhesive roots

Adhesive root types were found in the Anacardiaceae, Araceae, Cecropiaceae, Gesneriaceae, Hydrangeaceae, Moraceae, Pandanaceae, and Piperaceae (Fig. 3D) in Taiwan climbers. Approximately ten species, naming *Aeschynanthus acuminatus* (Gesneriaceae), *Dischidia formosana* (Apocynaceae), *Euonymus spraguei* (Celastraceae), *Euonymus trichocarpus* (Celastraceae), *Freycinetia formosana* (Pandanaceae), *Hedera rhombea* var. *formosana* (Araliaceae), *Parthenocissus tricuspidata* (Vitaceae) (Fig. 3J), *Poikilospermum acuminata* (Urticaceae), *Psychotria serpens* (Rubiaceae), and *Rhus ambigua* (Anacardiaceae) had adhesive roots. Among the 33 species in Apocynaceae, four had twining stems in the dextrorse combined with adhesive roots (Table 1).

Plants with adhesive roots can be firmly adsorbed onto the host because adhesive roots with root hairs will produce adhesive pads and can adsorb onto any substrate. For example, the adhesive roots of *Hedera helix* emit yellowish mucilage that is primarily composed of nanoparticles of arabinogalactan proteins and is high-strength adhesives (Huang et al. 2006). Therefore, the adhesive root groups can be firmly adsorbed onto the host using these mucilages.

Adhesive roots can climb trees of any diameter (Hegarty and Caballe 1991), and later successional forests composed of larger-diameter trees have more adhesive root species (Yang et al. 2018). Plants with twining stems generally prefer small-diameter trees, whereas those with adhesive roots prefer trees with larger diameters, which should also be further investigated.

#### 3. Hooks or grapnels

In this study, two *Calamus* (Arecaceae) and three *Uncaria* species (Rubiaceae) exhibited hooks or grapnels to scramble supports. Sperotto et al. (2020) proposed that the hooks or grapnels of these two genera are specialized structures separated from the simple scrambling type. The spiny of the *Calamus g*enus had three modifications in different positions: (a) sheaths covered with spiny; (b) flagella whip-like and armed with small grapnel-like spines, similar to an anchor-shaped terminal structure of three or more hooks; (c) branches and rachillae covered with clawed spines. The hooks of *Uncaria* genus originated from: (a) modified plagiotropic shoots into hooked spines (Ridsdale 1978); (b) modified peduncles into spines (Steyermark 1974), modified short shoots into thorns (Robbrecht 1988); and (c) modified branches into curved hooks (Sperotto et al. 2020). In the present study, the three *Uncaria* species (Fig. 3E) generally had paired, stiff, regular, and short spiral hooks derived from branches. The young plagiotropic branch of *A. hexapetalus* had two inflorescence hooks (Fig. 3F), each with one flower, and an older plagiotropic branch with two inflorescence hooks (Posluszny and Fisher 2000). Therefore, the spiny of *A. hexapetalus* generally had paired curved hooks derived from the inflorescence.

### Combination of two or three climber mechanisms

In Taiwan, 30 climbers exhibited a combination of two or three climbing methods. *Embelia laeta var. papilligera* exhibited simple scrambling combined with twining stem dextrorse and sinistrorse. Eight *Smilax* species, *S. arisanensis*,* S. bracteata* subsp. *bracteata, S. bracteata* subsp. *verruculosa*,* S. china*,* S. elongato-umbellata*,* S. horridiramula*,* S. ocreata*, and *S. sieboldii* had twining petiole duplication and simple scrambling. Six species, *Clerodendrum thomsoniae*, *Codonopsis kawakamii*,* Mikania cordata*, *Mikania micrantha*, *Reynoutria multiflorum* var. *hypoleuca,* *and* *Vernonia gratiosa* had both twining stem dextrorse and twining stem sinistrose, which is named neutral twining. Four species, *Hoya carnosa*,* Trachelospermum formosanum*,* T. gracilipes*,* T. jasminoides*, and *T. lanyuense*, had twining stems in the dextrorse and adhesive roots. Four species, *Asparagus cochinchinensis, Dioscorea collettii, D. cumingii,* and *D. esculenta* var. *spinosa*, had twining stems in sinistrorse and simple scrambling. Three species, *Parthenocissus tricuspidata*, *Tetrastigma obtectum*, and *T. obtectum* var. *glabrum* (Fig. 3G, H), had adhesive roots and twining peduncles or inflorescence. Two species, *Dioscorea cirrhosa* and *Quisqualis indica*, had twining stems in dextrorse and simple scrambling. One species, *Trichosanthes homophylla* had adhesive roots and twining modified shoot, and one species, *Artabotrys hexapetalus* had hooks or grapnels and simple scrambling.

### Climbing methods of Taiwan climbers in each order

We compared the climbing methods of orders/families reported by Sousa-Baena et al. (2018) and Sperotto et al. (2020), and the results are shown in Table 5. There were seven predominant climbing methods of Gentianales: twining stems (dextral and sinistral), twining modified shoot, simple scrambling, adhesive roots, hooks or grapnels, and a combination of adhesive roots and twining stems (dextral). These types differed from those in previous reports, except that the twining stems (dextral) were the same. Fabales had six types, and among them, only whole leaves modified into tendrils were not found in Taiwan. The diverse climbing habits in Gentianales and Fabales are more than those of Fabales and Asterales (Sousa-Baena et al. 2018). Rosales had five types in six families, and among them, prehensile branches were the same as reported in previous papers, and twining tendrils were not found in Taiwan. Some orders had specified climbing methods, such as Austrobaileyales and Dipsacales with twining stems (sinistral), Myrtales and Proteales with twining stems (dextral), Arecales, Brassicales, Malvales, and Santalales with simple scrambling, and Cornales with adhesive roots, which were not found in previous reports. In summary, Taiwan climbers had diverse climbing strategies available for climber dispersion and migration.

**Table 5:** List of orders and families of Taiwan climbers that possess climbing strategies

Order	CS (this study)	Family	CS (this study)	Sousa-Baena et al. (2018)	Sperotto et al. (2020)
Alismatales	AR	Araceae	AR	–	AR
Apiales	SS,AR	Araliaceae	SS,AR	–	–
Arecales	HG	Arecaceae	HG	–	HG
Asparagales	SS + TSs	Asparagaceae	SS + TSs	TLt	–
Asterales	TSd + TSs,TSd,SS	Asteraceae	TSd + TSs,TSd,SS	TLp,TTL,TLt,TLm	SS,TLp
		Campanulaceae	TSd + TSs,TSd	TLp	–
Austrobaileyales	TSs	Schisandraceae	TSs	–	–
Boraginales	SS	Heliotropiaceae	SS	–	–
Brassicales	SS	Capparaceae	SS	–	–
Caryophyllales	SS,TSd,TPI,	Amaranthaceae	SS	–	–
	TSd + TSs	Basellaceae	TSd	–	–
		Nyctaginaceae	SS	–	–
		Polygonaceae	TPI,SS,TSd + TSs	TPI,TMS	–
Celastrales	TSd,AR	Celastraceae	TSd,AR	TMS	PB
Cornales	AR	Hydrangeaceae	AR	–	–
Crossosomatales	SS	Stachyuraceae	SS	–	–
Cucurbitales	TMS	Cucurbitaceae	TMS	SSC,TMS,TMS + AR	TT
Dioscoreales	TSd,TSs, TSd + SS,TSs + SS	Dioscoreaceae	TSd,TSs,TSd + SS, TSs + SS	–	–
Dipsacales	TSs	Caprifoliaceae	TSs	–	–
Ericales	SS + TSd + TSs, TSd,TSs	Actinidiaceae	TSd	–	–
		Primulaceae	SS + TSd + TSs, TSd,TSs	–	–
Fabales	TSd,SS, PB,TTL,TMS,TSs	Fabaceae	TSd,SS,PB, TTL,TMS,TSs	TTL,WLT,TMS	PB,SS,
Gentianales	TSd,AR,SS, AR + TSd,TSs, TMS,HG	Apocynaceae	TSd,AR, AR + TSd,	TPI,TIA,TWI	TSd,TPI
		Gentianaceae	TSs,SS	–	–
		Loganiaceae	TSd,TMS	TMS	TMS
		Rubiaceae	TSd,TSs,AR,	–	–
			SS,HG	–	HG
Lamiales	TSd,TTL,AR,SS,	Acanthaceae	TSd	–	–
	TSd + TSs	Bignoniaceae	TTL	TTL,TT	TT,TLp
		Gesneriaceae	AR	–	–
		Lamiaceae	SS,TSd + TSs	–	–
		Oleaceae	TSd	–	–
Laurales	TLp,TSd	Hernandiaceae	TLp	TLp	–
		Lauraceae	TSd	–	–
Liliales	TPD + SS,TPD	Smilacaceae	TPD + SS,TPD	TPD	TPD
Magnoliales	HG + SS,PB	Annonaceae	HG + SS,PB	PB	–
Malpighiales	SS,TSd,TPI	Euphorbiaceae	SS	–	TSd
		Malpighiaceae	TSd	–	TSd
		Passifloraceae	TPI	TPI	–
		Phyllanthaceae	SS	–	–
Malvales	SS	Malvaceae	SS	–	SS
Myrtales	TSd + SS	Combretaceae	TSd + SS	–	–
Oxalidales	PB	Connaraceae	PB	TMS	PB
Piperales	TSd,AR,SS	Aristolochiaceae	TSd	–	TSd
		Piperaceae	AR,SS	–	AR
Pandanales	TSd,AR	Pandanaceae	AR	–	–
		Stemonaceae	TSd	–	–
Poales	TLt	Flagellariaceae	TLt	TLt	–
Proteales	TSd	Sabiaceae	TSd	–	–
Ranunculales	TSd,TLp	Lardizabalaceae	TSd	–	–
		Menispermaceae	TSd	TLp	TSd
		Ranunculaceae	TLp	TLp	TLp
Rosales	TSs,SS,AR, TSd,PB	Cannabaceae	TSs	–	SS
		Elaeagnaceae	SS	–	–
		Moraceae	AR,SS,TSd	–	–
		Rhamnaceae	TSs,SS,PB	PB,TPI	TT
		Rosaceae	SS	–	–
		Urticaceae	AR	–	–
Santalales	SS	Opiliaceae	SS	–	–
Sapindales	AR,SS,TPI	Anacardiaceae	AR	–	–
		Rutaceae	SS	–	–
		Sapindaceae	TPI	TPI	TT
Solanales	TSd,TSs,TLp,SS	Convolvulaceae	TSd,SS	–	–
		Solanaceae	TSs,TLp	–	TLp,SS,AR
Vitales	AR,TPI, TPI + AR	Vitaceae	AR,TPI, TPI + AR	TPI,TLr,TPI + AR	TPI + AR

*CS* climbing strategies, *TS* twining stem, *TSd* twining stem in dextrorse, *TSs* twining stem in sinistrorse, *TMS* twining modified shoot/stem, *TTL* twining terminal leaflets, *TLt* twining leaf tip, *TPD* twining petiole duplication, *TLp* twining leaf petioles, *TPI* twining peduncles or inflorescence, *PB* prehensile branch (also called TB: twining lateral branch), *SS* simple scrambling, *AR* adhesive roots, or adhesive pads, *HG* hooks or grapnels, *TT* twining tendrils (including TMS, TTL, TLt, TPD), *TLm* twining prolonged leaf midrib, *TIA* twining inflorescence axis, *TWI* twining whole inflorescence, *SSC* stem-stipule complex, *WLT* whole leaf modified into tendril

Fabaceae had 85 species with six climbing types, and Rubiaceae had 18 species with five climbing types, indicating that the climbing methods of Rubiaceae might differ from those of Fabaceae. Approximately 46% of the twining stems of all climbing methods found in Taiwan were consistent with those of most regions worldwide (Nabe-Nielsen 2001; Reddy and Parthasarathy 2003; Senbeta et al. 2005). Among these species, *Artabotrys hexapetalus* had two climbing mechanisms, hooks/grapnels and simple scrambling. Most Taiwanese Vitaceae species have tendrils; among them, three species, *Parthenocissus tricuspidata*,* Tetrastigma obtectum* var. *glabrum*, and *Tetrastigma obtectum* var. *obtectum* also have adhesive roots. The remaining three *Tetrastigma* species within these genera are still under investigation. Among the seven *Trichosanthes* species in Taiwan, we determined that *Trichosanthes homophylla* exhibited a combination of modified stems (Fig. 3I) and adhesive roots. The remaining species must be observed in the near future.

Four reproductive and five vegetative organs of tendrils were identified (Table 2). Some vegetative organs, for example, prolonged midrib, prolonged forked tips of midribs, modified petioles, whole leaf modified into a simple tendril, and modified compound leaf rachis; or some reproductive organs: inflorescence lateral branches, inflorescence peduncles, and flower pedicels, were not found in this study. The information collected in the present study will provide fundamental evidence for further studies on climber development and survival mechanisms.

## Conclusion

In this study, we explored the climbing strategies of Taiwanese climbers and examined their organs of origin. The results revealed approximately 21 climbing methods, including a combination of two or three climbing methods. Approximately 46% of all climbers twined their stems, followed by simple scrambling and twining of tendrils. Most families used only one climbing method; however, Fabaceae had the highest number of climbing types (six), followed by Rubiaceae (five). Apocynaceae and Fabaceae plants had twining stems in dextrorse, and only *Alyxia taiwanensis* and *Wisteriopsis reticulata* were sinistrorse, showing that the twining stem type was good evidence for species identification. The prehensile branch of the *Fissi*s*tigma* genus, *Ventilago* genus, *Connarus subinaequifolius*, *Rourea minor*, and *Dalbergia benthamii* are derived from second-order or modified stems. Some modified tendrils in Cucurbitaceae, such as the modified stems-stipule complex of *Thladiantha* genus and modified stems with adhesive pads of the genera *Neoalsomitra* and *Trichosanthes*, were required for observation. Climbing methods for the genus *Tetrastigma* in Taiwan include a combination of inflorescence tendrils with adhesive roots, such as* T. obtectum* var. *glabrum* and *T. obtectum* var. *obtectum*. Whether the other three species have these characteristics should be investigated in future studies. *Artabotrys hexapetalus* has hooks or grapnels in younger stems and simple scrambling in older stems. This research on the climbing methods of Taiwan climbers will aid in establishing Taiwan climbers as common taxon-specific or planting configuration data. This information is crucial for future climber research to ensure the conservation of biodiversity.

### Supplementary Information

**Additional file 1: Appendix S1.** The checklist of climbing strategies for Taiwan climbers.

## Data Availability

Not applicable.
