# Supplementary material for: Climbing strategies of Taiwan climbers
Source: Bot Stud. 2023 Sep 22;64:26. doi: 10.1186/s40529-023-00399-4 (PMC10516820; doi:10.1186/s40529-023-00399-4)
Supplement: Supplementary file 1 — Additional file 1: Appendix S1. The checklist of climbing strategies for Taiwan climbers. [file 40529_2023_399_MOESM1_ESM.docx]

Appendix S1 The checklist of climbing strategies for Taiwan climbers

| Order | APG4 family | Species name | Climbing  Strategies |
| --- | --- | --- | --- |
| Lamiales | Acanthaceae | *Thunbergia alata* Bojer ex Sims | TSd |
| Lamiales | Acanthaceae | *Thunbergia fragrans*Roxb. | TSd |
| Lamiales | Acanthaceae | *Thunbergia grandiflora* Roxb. | TSd |
| Lamiales | Acanthaceae | *Thunbergia gregorii* S. Moore | TSd |
| Lamiales | Acanthaceae | *Thunbergia laurifolia*Lindl. | TSd |
| Ericales | Actinidiaceae | *Actinidia arguta* (Sieb. & Zucc.) Planch. ex Miquel | TSd |
| Ericales | Actinidiaceae | *Actinidia callosa* var. *discolor* C. F. Liang Feng | TSd |
| Ericales | Actinidiaceae | *Actinidia latifolia* (Gardner & Champ.) Merr. | TSd |
| Ericales | Actinidiaceae | *Actinidia rufa* (Sieb. & Zucc.) Planch. ex Miquel | TSd |
| Ericales | Actinidiaceae | *Actinidia setosa* (H. L. Li) C. F. Liang & A. R. Ferguson* | TSd |
| Caryophyllales | Amaranthaceae | *Deeringia amaranthoides* | SS |
| Sapindales | Anacardiaceae | *Rhus ambigua* Lav. ex Dippel | AR |
| Magnoliales | Annonaceae | *Artabotrys hexapetalus* (Linnaeus f.) Bhandari | HG,SS |
| Magnoliales | Annonaceae | *Fissistigma glaucescens* (Hance) Merr. | PB |
| Magnoliales | Annonaceae | *Fissistigma oldhamii* (Hemsl.) Merr. | PB |
| Gentianales | Apocynaceae | *Alyxia sibuyanensis* Elmer | TSd |
| Gentianales | Apocynaceae | *Alyxia sinensis* Champ. ex Benth. | TSd |
| Gentianales | Apocynaceae | *Alyxia taiwanensis* S. Y. Lu & Yuen P. Yang* | TSs |
| Gentianales | Apocynaceae | *Anodendron affine* (Hook. & Arn.) Druce | TSd |
| Gentianales | Apocynaceae | *Anodendron benthamiana* Hemsl.* | TSd |
| Gentianales | Apocynaceae | *Cryptolepis sinensis* (Lour.) Merr. | TSd |
| Gentianales | Apocynaceae | *Cynanchum boudieri* H. Lév. & Vaniot | TSd |
| Gentianales | Apocynaceae | *Cynanchum formosanum*(Maxim.) Hemsl. ex Forbes & Hemsl.* | TSd |
| Gentianales | Apocynaceae | *Cynanchum lanhsuense* T. Yamaz.* | TSd |
| Gentianales | Apocynaceae | *Dischidia formosana* Maxim.* | AR |
| Gentianales | Apocynaceae | *Dregea volubilis* (L. f.) Benth. | TSd |
| Gentianales | Apocynaceae | *Gymnema sylvestre* (Retz.) Schultes | TSd |
| Gentianales | Apocynaceae | *Heterostemma brownii* Hayata | TSd |
| Gentianales | Apocynaceae | *Hoya carnosa* (L.f.) R.Br. | AR,TSd |
| Gentianales | Apocynaceae | *Jasminanthes mucronata* (Blanco) W. D. Stevens & P. T. Li | TSd |
| Gentianales | Apocynaceae | *Marsdenia formosana* Masam. | TSd |
| Gentianales | Apocynaceae | *Marsdenia tinctoria* R. Br. | TSd |
| Gentianales | Apocynaceae | *Melodinus angustifolius* Hayata* | TSd |
| Gentianales | Apocynaceae | *Parsonsia alboflavescens*(Dennst.) Mabb. | TSd |
| Gentianales | Apocynaceae | *Trachelospermum asiaticum* (Sieb. & Zucc.) Nakai | AR,TSd |
| Gentianales | Apocynaceae | *Trachelospermum jasminoides* (Lindl.) Lemaire | AR,TSd |
|  | Apocynaceae | *Trachelospermum lucidum* (D. Don) K.Schum. | AR,TSd |
| Gentianales | Apocynaceae | *Urceola micrantha* (Wallich *ex* G. Don) D. J. Middleton | TSd |
| Gentianales | Apocynaceae | *Urceola rosea* (Hook. & Arn.) D. J. Middleton | TSd |
| Gentianales | Apocynaceae | *Vincetoxicum brownii* (Hayata) Meve & Liede | TSd |
| Gentianales | Apocynaceae | *Vincetoxicum chinense* Moore | TSd |
| Gentianales | Apocynaceae | *Vincetoxicum fordii* (Hemsl.) Kuntze | TSd |
| Gentianales | Apocynaceae | *Vincetoxicum hirsutum* (Wallich) Kuntze | TSd |
| Gentianales | Apocynaceae | *Vincetoxicum insulicola* Meve & Liede* | TSd |
| Gentianales | Apocynaceae | *Vincetoxicum lui* (Y.H. Tseng & C.T. Chao) Meve & Liede* | TSd |
| Gentianales | Apocynaceae | *Vincetoxicum oshimae* (Hayata) Meve & Liede* | TSd |
| Gentianales | Apocynaceae | *Vincetoxicum sui* (Y.H. Tseng & C.T. Chao) T.C. Hsu* | TSd |
| Gentianales | Apocynaceae | *Vincetoxicum taiwanense* (Hatus.) T.C. Hsu* | TSd |
| Alismatales | Araceae | *Epipremnum aureum* (Linden & André) G.S.Bunting | AR |
| Alismatales | Araceae | *Epipremnum pinnatum* (L.) Engl. ex Engl. & Kraus | AR |
| Alismatales | Araceae | *Pothoidium lobbianum* Schott | AR |
| Alismatales | Araceae | *Pothos chinensis* (Raf.) Merr. | AR |
| Alismatales | Araceae | *Rhaphidophora hongkongensis* Schott | AR |
| Alismatales | Araceae | *Rhaphidophora liukiuensis* Hatus. | AR |
| Alismatales | Araceae | *Syngonium angustatum* Schott | AR |
| Alismatales | Araceae | *Syngonium podophyllum* Schott | AR |
| Apiales | Araliaceae | *Eleutherococcus trifoliatus* (L.) S. Y. Hu var. *setusus* (H. L. Li) H. Ohashi | SS |
| Apiales | Araliaceae | *Eleutherococcus trifoliatus* (L.) S. Y. Hu var. *trifoliatus* | SS |
| Apiales | Araliaceae | *Hedera rhombea* (Miq.) Bean var. *formosana* (Nakai) H. L. Li* | AR |
| Arecales | Arecaceae | *Calamus formosanus* Becc.* | HG |
| Arecales | Arecaceae | *Calamus siphonospathus* Martius* | HG |
| Piperales | Aristolochiaceae | *Aristolochia cucurbitifolia* Hayata*** | TSd |
| Piperales | Aristolochiaceae | *Aristolochia foveolata* Merr. (kao) | TSd |
| Piperales | Aristolochiaceae | *Aristolochia shimadae* Hayata | TSd |
| Piperales | Aristolochiaceae | *Aristolochia yujungiana* C.T.Lu & J.C.Wang | TSd |
| Piperales | Aristolochiaceae | *Aristolochia zollingeriana* Miq. | TSd |
| Asparagales | Asparagaceae | *Asparagus cochinchinensis* (Lour.) Merr. | SS,TSs |
| Asterales | Asteraceae | *Blumea riparia* (Blume) DC. var. *megacephala* Randeria, | SS |
| Asterales | Asteraceae | *Microglossa pyrifolia* (Lam.) Kuntze | SS |
| Asterales | Asteraceae | *Mikania cordata* (Burm. f.) B. L. Rob. | TSd,TSs |
| Asterales | Asteraceae | *Mikania micrantha* Kunth | TSd,TSs |
| Asterales | Asteraceae | *Senecio scandens* Buchanan-Hamilton ex D. Don | SS |
| Asterales | Asteraceae | *Vernonia elliptica* DC | SS |
| Asterales | Asteraceae | *Vernonia gratiosa* Hance | TSd,TSs |
| Asterales | Asteraceae | *Wedelia biflora* (L.) DC. | SS |
| Caryophyllales | Basellaceae | *Anredera cordifolia* (Tenore) van Steenis | TSd |
| Caryophyllales | Basellaceae | *Basella alba* L. | TSd |
| Lamiales | Bignoniaceae | *Anemopaegma chamberlaynii* (Sims) Bureau & K.Schum. | TTL |
| Lamiales | Bignoniaceae | *Pyrostegia venusta* (Ker-Gawl.) Miers | TTL |
| Asterales | Campanulaceae | *Codonopsis javanica* (Blume) Miq. subsp. *japonica* (Maxim. ex Makino) Lammers | TSd |
| Asterales | Campanulaceae | *Codonopsis kawakamii* Hayata* | TSd,TSs |
| Rosales | Cannabaceae | *Humulus scandens* (Lour.) Merr. | TSs |
| Brassicales | Capparaceae | *Capparis acutifolia* Sweet | SS |
| Brassicales | Capparaceae | *Capparis floribunda* Wight | SS |
| Brassicales | Capparaceae | *Capparis formosana* Hemsl.* | SS |
| Brassicales | Capparaceae | *Capparis lanceolaris* DC. | SS |
| Brassicales | Capparaceae | *Capparis pubiflora* DC. | SS |
| Dipsacales | Caprifoliaceae | *Lonicera acuminata* Wall. | TSs |
| Dipsacales | Caprifoliaceae | *Lonicera apodantha* Ohwi* | TSs |
| Dipsacales | Caprifoliaceae | *Lonicera hypoglauca* Miq. | TSs |
| Dipsacales | Caprifoliaceae | *Lonicera japonica* Thunb. | TSs |
| Dipsacales | Caprifoliaceae | *Lonicera macrantha* (D. Don) Spreng. | TSs |
| Celastrales | Celastraceae | *Celastrus hindsii* Benth. | TSd |
| Celastrales | Celastraceae | *Celastrus kusanoi* Hayata | TSd |
| Celastrales | Celastraceae | *Celastrus paniculatus* Willd. | TSd |
| Celastrales | Celastraceae | *Celastrus punctatus* Thunb. | TSd |
| Celastrales | Celastraceae | *Euonymus spraguei* Hayata* | AR |
| Celastrales | Celastraceae | *Euonymus trichocarpus* Hayata* | AR |
| Celastrales | Celastraceae | *Tripterygium wilfordii* Hook. f. | TSd |
| Myrtales | Combretaceae | *Quisqualis indica* L. | TSd,SS |
| Oxalidales | Connaraceae | *Connarus subinaequifolius* Elmer | PB |
| Oxalidales | Connaraceae | *Rourea minor* (Gaertn.) Leenhouts | PB |
| Solanales | Convolvulaceae | *Argyreia akoensis*S. Z. Yang, P. H. Chen & G. W. Staples* | TSd |
| Solanales | Convolvulaceae | *Argyreia formosana* Ishigami ex T. Yamaz.* | TSd |
| Solanales | Convolvulaceae | *Camonea umbellata* (L.) A.R.Simões & Staples | TSd |
| Solanales | Convolvulaceae | *Camonea vitifolia* (Burm.f.) A.R.Simões & Staples | TSd |
| Solanales | Convolvulaceae | *Cuscuta australis* R.Br. | TSd |
| Solanales | Convolvulaceae | *Cuscuta campestris* Yunck. | TSd |
| Solanales | Convolvulaceae | *Cuscuta chinensis* Lam. | TSd |
| Solanales | Convolvulaceae | *Cuscuta japonica* Choisy var. *formosana* (Hayata) Yunck.* | TSd |
| Solanales | Convolvulaceae | *Cuscuta japonica* Choisy var. *japonica* | TSd |
| Solanales | Convolvulaceae | *Decalobanthus similis* (Elmer) A.R.Simões & Staples | TSd |
| Solanales | Convolvulaceae | *Dichondra micrantha* Urb. | SS |
| Solanales | Convolvulaceae | *Distimake cissoides* (Lam.) A.R.Simões & Staples | TSd |
| Solanales | Convolvulaceae | *Distimake dissectus* (Jacq.) A.R.Simões & Staples | TSd |
| Solanales | Convolvulaceae | *Distimake quinatus* (R.Br.) A.R.Simões & Staples | TSd |
| Solanales | Convolvulaceae | *Distimake quinquefolius* (L.) A.R.Simões & Staples | TSd |
| Solanales | Convolvulaceae | *Distimake tuberosus* (L.) A.R.Simões & Staples | TSd |
| Solanales | Convolvulaceae | *Erycibe henryi* Prain | TSd |
| Solanales | Convolvulaceae | *Evolvulus nummularius* (L.) L. | SS |
| Solanales | Convolvulaceae | *Hewittia malabarica* (L.) Suresh | TSd |
| Solanales | Convolvulaceae | *Ipomoea alba* L. | TSd |
| Solanales | Convolvulaceae | *Ipomoea aquatica* Forssk. | TSd |
| Solanales | Convolvulaceae | *Ipomoea batatas* (L.) Lam. | TSd |
| Solanales | Convolvulaceae | *Ipomoea biflora* (L.) Persoon | TSd |
| Solanales | Convolvulaceae | *Ipomoea cairica* (L.) Sweet | TSd |
| Solanales | Convolvulaceae | *Ipomoea carnea* Jacq. subsp*. fistulosa* (Mart. ex Choisy) D. F. Austin | TSd |
| Solanales | Convolvulaceae | *Ipomoea cholulensis* Kunth | TSd |
| Solanales | Convolvulaceae | *Ipomoea eriocarpa* R.Br. | TSd |
| Solanales | Convolvulaceae | *Ipomoea hederacea* (L.) Jacq. | TSd |
| Solanales | Convolvulaceae | *Ipomoea hederifolia* L. | TSd |
| Solanales | Convolvulaceae | *Ipomoea imperati* (Vahl) Griseb | SS |
| Solanales | Convolvulaceae | *Ipomoea indica* (Burm. f.) Merr. | TSd |
| Solanales | Convolvulaceae | *Ipomoea lacunosa* L. | TSd |
| Solanales | Convolvulaceae | *Ipomoea leucantha* Jacq. | TSd |
| Solanales | Convolvulaceae | *Ipomoea littoralis* Blume | TSd |
| Solanales | Convolvulaceae | *Ipomoea mauritiana* Jacq. | TSd |
| Solanales | Convolvulaceae | *Ipomoea nil* (L.)Roth. | TSd |
| Solanales | Convolvulaceae | *Ipomoea obscura* (L.) Ker Gawl. | TSd |
| Solanales | Convolvulaceae | *Ipomoea pes-caprae* (L.) R. Br. subsp.  *brasiliensis* (L.) Oostst. | SS |
| Solanales | Convolvulaceae | *Ipomoea pes-tigridis* L. | TSd |
| Solanales | Convolvulaceae | *Ipomoea purpurea* (L.) Roth. | TSd |
| Solanales | Convolvulaceae | *Ipomoea quamoclit* L. | TSd |
| Solanales | Convolvulaceae | *Ipomoea sumatrana* (Blume) Oostst. | TSd |
| Solanales | Convolvulaceae | *Ipomoea trifida* (Kunth) G. Don | TSd |
| Solanales | Convolvulaceae | *Ipomoea triloba* L. | TSd |
| Solanales | Convolvulaceae | *Ipomoea violacea* L. | TSd |
| Solanales | Convolvulaceae | *Ipomoea wrightii* A. Gray | TSd |
| Solanales | Convolvulaceae | *Jacquemontia paniculata* (Burm. f.) Hallier f. | TSd |
| Solanales | Convolvulaceae | *Jacquemontia polyantha* (Schltdl. & Cham.) Hallier f. | TSd |
| Solanales | Convolvulaceae | *Jacquemontia tamnifolia* (L.)Griseb. | TSd |
| Solanales | Convolvulaceae | *Lepistemon binectariferum* (Wall.) Kuntze  var. *trichocarpum* (Gagnep.) Ooststr. | TSd |
| Solanales | Convolvulaceae | *Lepistemon intermedius* Hallier f. | TSd |
| Solanales | Convolvulaceae | *Merremia gemella* (Burm.f.) Hallier f. | TSd |
| Solanales | Convolvulaceae | *Merremia hederacea* (Burm.f.) Hallier f. | TSd |
| Solanales | Convolvulaceae | *Merremia hirta* (L.) Merr. | TSd |
| Solanales | Convolvulaceae | *Operculina turpethum* (L.) S. Manso | TSd |
| Solanales | Convolvulaceae | *Stictocardia tilifolia* (Desr.) Hallier f. | TSd |
| Solanales | Convolvulaceae | *Xenostegia tridentata* (L.) D. F. Austin & Staples | TSd |
| Cucurbitales | Cucurbitaceae | *Actinostemma tenerum* Griff. | TMS |
| Cucurbitales | Cucurbitaceae | *Benincasa hispida* (Thunberg) Cogniaux | TMS |
| Cucurbitales | Cucurbitaceae | *Citrullus lanatus* (Thunberg) Matsumura & Nakai | TMS |
| Cucurbitales | Cucurbitaceae | *Coccinia grandis* (L.) Voigt | TMS |
| Cucurbitales | Cucurbitaceae | *Cucumis melo* Linnaeus subsp. *melo* | TMS |
| Cucurbitales | Cucurbitaceae | *Cucurbita moschata* Duchesne | TMS |
| Cucurbitales | Cucurbitaceae | *Diplocyclos palmatus* (L.) C. Jeffrey | TMS |
| Cucurbitales | Cucurbitaceae | *Gynostemma laxum* (Wall.) Cogn. | TMS |
| Cucurbitales | Cucurbitaceae | *Gynostemma pentaphllum* (Thunb.) Makino | TMS |
| Cucurbitales | Cucurbitaceae | *Gynostemma simplicifolium* Bl. | TMS |
| Cucurbitales | Cucurbitaceae | *Luffa aegyptiaca* Miller | TMS |
| Cucurbitales | Cucurbitaceae | *Melothria pendula* L. | TMS |
| Cucurbitales | Cucurbitaceae | *Momordica charantia* L. | TMS |
| Cucurbitales | Cucurbitaceae | *Momordica charantia* L. var. *abbreviata* Ser. | TMS |
| Cucurbitales | Cucurbitaceae | *Momordica cochinchinensis* (Lour.) Spreng. | TMS |
| Cucurbitales | Cucurbitaceae | *Mukia maderaspatana* (L.) M.J. Roem | TMS |
| Cucurbitales | Cucurbitaceae | *Neoalsomitra integrifolia* (Cogn.) Hutch. | TMS |
| Cucurbitales | Cucurbitaceae | *Sechium edule* (Jacq.) Sw. | TMS |
| Cucurbitales | Cucurbitaceae | *Sicyos angulatus* L. | TMS |
| Cucurbitales | Cucurbitaceae | *Sinobaijiania taiwaniana* (Hayata) C. Jeffrey & W. J. de Wilde* | TMS |
| Cucurbitales | Cucurbitaceae | *Solena heterophylla* Lour. | TMS |
| Cucurbitales | Cucurbitaceae | *Thladiantha nudiflora* Hemsl. ex Forbes & Hemsl. | TMS |
| Cucurbitales | Cucurbitaceae | *Thladiantha punctata* Hayata | TMS |
| Cucurbitales | Cucurbitaceae | *Trichosanthes costata* Blume | TMS |
| Cucurbitales | Cucurbitaceae | *Trichosanthes cucumeroides (*Ser.) Maxim. ex Franch. & Sav. | TMS |
| Cucurbitales | Cucurbitaceae | *Trichosanthes homophylla* Hayata | TMS,AR |
| Cucurbitales | Cucurbitaceae | *Trichosanthes laceribracteata* Hayata | TMS |
| Cucurbitales | Cucurbitaceae | *Trichosanthes ovigera* Blume | TMS |
| Cucurbitales | Cucurbitaceae | *Trichosanthes quinquangulata* A. Gray | TMS |
| Cucurbitales | Cucurbitaceae | *Trichosanthes rosthornii* Harms | TMS |
| Cucurbitales | Cucurbitaceae | *Zehneria japonica* (Thunb.) H.Y.Liu | TMS |
| Cucurbitales | Cucurbitaceae | *Zehneria mucronata* (Blume) Miq. | TMS |
| Dioscoreales | Dioscoreaceae | *Dioscorea alata* L. | TSd |
| Dioscoreales | Dioscoreaceae | *Dioscorea batatas* Decne. | TSd |
| Dioscoreales | Dioscoreaceae | *Dioscorea benthamii* Prain & Burkill | TSd |
| Dioscoreales | Dioscoreaceae | *Dioscorea bulbifera* L. | TSs |
| Dioscoreales | Dioscoreaceae | *Dioscorea cirrhosa* Lour. | TSd, SS |
| Dioscoreales | Dioscoreaceae | *Dioscorea codonopsidifolia* Kamik.* | TSs |
| Dioscoreales | Dioscoreaceae | *Dioscorea collettii* Hook. f. | TSs, SS |
| Dioscoreales | Dioscoreaceae | *Dioscorea cumingii* Prain & Burkill | TSs, SS |
| Dioscoreales | Dioscoreaceae | *Dioscorea doryphora* Hance | TSd |
| Dioscoreales | Dioscoreaceae | *Dioscorea esculenta* (Lour.) Burkill var. *spinosa* R.Knuth | TSs, SS |
| Dioscoreales | Dioscoreaceae | *Dioscorea hispida* Dennst. | TSs |
| Dioscoreales | Dioscoreaceae | *Dioscorea japonica* Thunb. var. *japonica* | TSd |
| Dioscoreales | Dioscoreaceae | *Dioscorea persimilis* Prain & Burkill | TSd |
| Dioscoreales | Dioscoreaceae | *Dioscorea sansibarensis* Pax | TSs |
| Rosales | Elaeagnaceae | *Elaeagnus formosana* Nakai* | SS |
| Rosales | Elaeagnaceae | *Elaeagnus formosensis* Hatusima* | SS |
| Rosales | Elaeagnaceae | *Elaeagnus glabra* Thunb. | SS |
| Rosales | Elaeagnaceae | *Elaeagnus grandifolia* Hayata* | SS |
| Rosales | Elaeagnaceae | *Elaeagnus macrophylla* Thunb.* | SS |
| Rosales | Elaeagnaceae | *Elaeagnus thunbergii* Serv.* | SS |
| Rosales | Elaeagnaceae | *Elaeagnus triflora* Roxb. | SS |
| Malpighiales | Euphorbiaceae | *Mallotus repandus* (Willd.) Müll. Arg. | SS |
| Fabales | Fabaceae | *Abrus precatorius* L. | TSd |
| Fabales | Fabaceae | *Amphicarpaea bracteata* (L.) Fernald var.  *japonica* (Oliver) Ohashi. | TSd |
| Fabales | Fabaceae | *Apios taiwaniana* Hosok.* | TSd |
| Fabales | Fabaceae | *Caesalpinia bonduc* (L.) Roxb. | SS |
| Fabales | Fabaceae | *Caesalpinia crista* L. | SS |
| Fabales | Fabaceae | *Caesalpinia decapetala* (Roth) Alston | SS |
| Fabales | Fabaceae | *Caesalpinia minax* Hance | SS |
| Fabales | Fabaceae | *Cajanus scarabaeoides* (L.) du Petit-Thouars | TSd |
| Fabales | Fabaceae | *Callerya nitida* (Benth.) R. Geesink | TSd |
| Fabales | Fabaceae | *Calopogonium mucunoides* Desv. | TSd |
| Fabales | Fabaceae | *Canavalia cathartica* Thouars | TSd |
| Fabales | Fabaceae | *Canavalia ensiformis* (L.) DC. | TSd |
| Fabales | Fabaceae | *Canavalia lineata* (Thunb.) DC. | TSd |
| Fabales | Fabaceae | *Canavalia rosea* (Sw.) DC. | TSd |
| Fabales | Fabaceae | *Centrosema plumieri* (Turp. et Pers.) Benth. | TSd |
| Fabales | Fabaceae | *Centrosema pubescens* Benth. | TSd |
| Fabales | Fabaceae | *Clitoria falcata* Lam. | TSd |
| Fabales | Fabaceae | *Clitoria ternatea* L. | TSd |
| Fabales | Fabaceae | *Dalbergia benthamii* Prain | PB |
| Fabales | Fabaceae | *Derris laxiflora* Benth.* | TSd |
| Fabales | Fabaceae | *Derris taiwaniana* (Hayata) Z.Q. Song | TSd |
| Fabales | Fabaceae | *Derris trifoliata* Lour. | TSd |
| Fabales | Fabaceae | *Dolichos rilobus* L.var. *kosyunensis* (Hosok.) H. Ohashi & Tateishi* | TSd |
| Fabales | Fabaceae | *Dolichovigna pilosa* (Willd.) Niyomdham | TSd |
| Fabales | Fabaceae | *Dumasia truncata* Siebold & Zucc. | TSd |
| Fabales | Fabaceae | *Dumasia villosa* DC. subsp. *bicolor* (Hayata) Ohashi & Tateishi * | TSd |
| Fabales | Fabaceae | *Dunbaria merrillii* Elmer | TSd |
| Fabales | Fabaceae | *Dunbaria rotundifolia* (Lour.) Merr. | TSd |
| Fabales | Fabaceae | *Dunbaria villosa* (Thunb.) Makino | TSd |
| Fabales | Fabaceae | *Entada phaseoloides* (L.) Merr. subsp. *phaseoloides* | TTL |
| Fabales | Fabaceae | *Entada phaseoloides* (L.) Merr. subsp. *tonkinensis* (Gagnep.) H. Ohashi | TTL |
| Fabales | Fabaceae | *Entada rheedei* Spreng. | TTL |
| Fabales | Fabaceae | *Galactia tashiroi* Maxim. | TSd |
| Fabales | Fabaceae | *Galactia tenuiflora* (Klein ex Willd.) Wight & Arn. | TSd |
| Fabales | Fabaceae | *Galactia tenuiflora* (Klein ex Willd.) Wight & Arn. var. *villosa* (Wight & Arn.) Baker | TSd |
| Fabales | Fabaceae | *Glycine dolichocarpa* Tateishi & H. Ohashi* | TSd |
| Fabales | Fabaceae | *Glycine max* (L.) Merr. subsp. *formosana* (Hosok.) Tateishi & H. Ohashi* | TSd |
| Fabales | Fabaceae | *Glycine max* (L.) Merr. subsp. *max* | TSd |
| Fabales | Fabaceae | *Glycine tabacina* (Labille) Bentham | TSd |
| Fabales | Fabaceae | *Glycine tomentella* Hayata | TSd |
| Fabales | Fabaceae | *Lablab purpureus* (L.)Sweet | TSd |
| Fabales | Fabaceae | *Leptospron adenanthum* (G.F. Meyer) A. Delgado | TSd |
| Fabales | Fabaceae | *Macroptilium atropurpureum* (DC.) Urb. | TSd |
| Fabales | Fabaceae | *Macroptilium bracteatum* (Nees & Mart.) Maréchal & Baudet | TSd |
| Fabales | Fabaceae | *Macroptilium lathyroides* (L.) Urb. | TSd |
| Fabales | Fabaceae | *Macrotyloma axillare* (E. Mey.) Verdc. | TSd |
| Fabales | Fabaceae | *Macrotyloma uniflorum* Lam. | TSd |
| Fabales | Fabaceae | *Mimosa diplotricha* C. Wright ex Sauvalle | SS |
| Fabales | Fabaceae | *Mucuna gigantea* (Willd.) DC.subsp. *tashiroi* (Hayata) H. Ohashi & Tateishi* | TSd |
| Fabales | Fabaceae | *Mucuna macrocarpa* Wall. | TSd |
| Fabales | Fabaceae | *Mucuna membranacea* Hayata | TSd |
| Fabales | Fabaceae | *Mucuna pruriens* (L.) DC. var. *pruriens* | TSd |
| Fabales | Fabaceae | *Mucuna pruriens* (L.) DC. var. *utilis* (Wall. ex Wight) Burck | TSd |
| Fabales | Fabaceae | *Neonotonia wightii* (Wight & Arn.) Lackey | TSd |
| Fabales | Fabaceae | *Pachyrhizus erosu* (L.)Urb. | TSd |
| Fabales | Fabaceae | *Paraderris canarensis* (Dalzell) Adema | TSd |
| Fabales | Fabaceae | *Paraderris elliptica* (Wallich) Adema | TSd |
| Fabales | Fabaceae | *Phanera championii* Benth. | TMS |
| Fabales | Fabaceae | *Psophocarpus tetragonolobus* (L.) DC. | TSd |
| Fabales | Fabaceae | *Pueraria lobata* (Willd.) Ohwi subsp. *thomsonii* (Benth.) Ohashi & Tateishi | TSd |
| Fabales | Fabaceae | *Pueraria montana* (Lour.) Merr. | TSd |
| Fabales | Fabaceae | *Pueraria phaseoloides* (Roxb.) Benth. var. *javanica* (Benth.) Baker | TSd |
| Fabales | Fabaceae | *Pueraria phaseoloides* (Roxb.) Benth. var. *phaseoloides* | TSd |
| Fabales | Fabaceae | *Rhynchosia minima* (L.) DC. | TSd |
| Fabales | Fabaceae | *Rhynchosia rothii* Benth. ex Aitch. | TSd |
| Fabales | Fabaceae | *Rhynchosia volubilis* Lour. | TSd |
| Fabales | Fabaceae | *Senegalia caesia* (L.) Maslin, Seigler & Ebinger | SS |
| Fabales | Fabaceae | *Teramnus labialis* (L. f.) Spr. | TSd |
| Fabales | Fabaceae | *Vicia cracca* L. | TTL |
| Fabales | Fabaceae | *Vicia hirsuta* (L.) S. F. Gray | TTL |
| Fabales | Fabaceae | *Vicia sativa* L. subsp. *nigra* (L.) Ehrh. | TTL |
| Fabales | Fabaceae | *Vicia tetrasperma* (L.) Moench | TTL |
| Fabales | Fabaceae | *Vicia villosa* Roth subsp. *varia* (Host) Corbière | TTL |
| Fabales | Fabaceae | *Vigna angularis* (Willd.) Ohwi & H. Ohashi var. *nipponensis* (Ohwi) Ohwi & H. Ohashi | TSd |
| Fabales | Fabaceae | *Vigna hosei* (Craib) Backer | TSd |
| Fabales | Fabaceae | *Vigna luteola* (Jacq.) Benth. | TSd |
| Fabales | Fabaceae | *Vigna marina* (Burm.) Merr. | TSd |
| Fabales | Fabaceae | *Vigna radiata* (L.) Wilczek var. *radiata* | TSd |
| Fabales | Fabaceae | *Vigna radiata* (L.) Wilczek var. *sublobata* (Roxb.) Verdc. | TSd |
| Fabales | Fabaceae | *Vigna reflexopilos* Hayata | TSd |
| Fabales | Fabaceae | *Vigna riukiuensis* (Ohwi) Ohwi & H. Ohashi | TSd |
| Fabales | Fabaceae | *Vigna umbellata* (Thunb.) Ohwi & H. Ohashi | TSd |
| Fabales | Fabaceae | *Vigna vexillata* (L.) A. Rich. var. *tsusimensis* Matsum. | TSd |
| Fabales | Fabaceae | *Wisteriopsis reticulata* (Benth.) J.Compton & Schrire | TSs |
| Poales | Flagellariaceae | *Flagellaria indica* L. | TLt |
| Gentianales | Gentianaceae | *Pterygocalyx volubilis* Maxim. | TSs |
| Gentianales | Gentianaceae | *Tripterospermum alutaceifolium* (T. S. Liu & Chiu C. Kuo) J. Murata* | TSs |
| Gentianales | Gentianaceae | *Tripterospermum hualienense* T. C. Hsu & S. W. Chung* | TSs |
| Gentianales | Gentianaceae | *Tripterospermum lanceolatum* (Hayata) H. Hara ex Satake* | TSs |
| Gentianales | Gentianaceae | *Tripterospermum lilungshanensis* C. H. Chen, J .C. Wang & Y. C. Chang* | TSs |
| Gentianales | Gentianaceae | *Tripterospermum luzonense* (Vidal) J. Murata | TSs |
| Gentianales | Gentianaceae | *Tripterospermum taiwanense*(Masam.) Satake* | TSs |
| Gentianales | Gentianaceae | *Tripterospermum cordifolium* (Yamam.) Satake | SS |
| Gentianales | Gentianaceae | *Tripterospermum microphyllum* Harry Sm. | SS |
| Lamiales | Gesneriaceae | *Aeschynanthus acuminatus* Wall. ex A. DC. | AR |
| Boraginales | Heliotropiaceae | *Heliotropium sarmentosum* (Lam.) Craven | SS |
| Laurales | Hernandiaceae | *Illigera luzonensis* (C. Presl) Merr. | TLp |
| Cornales | Hydrangeaceae | *Hydrangea anomala* D. Don | AR |
| Cornales | Hydrangeaceae | *Hydrangea fauriei* (Hayata) Y. De Smet & Granados | AR |
| Cornales | Hydrangeaceae | *Hydrangea integrifolia* Hayata | AR |
| Cornales | Hydrangeaceae | *Hydrangea viburnoides* (Hook. f. & Thomson) Y. De Smet  & Granados var*. parviflora* Oliv. ex Maxim. | AR |
| Laurales | Lamiaceae | *Clerodendrum thomsoniae* Balf.f. | TSs,TSd |
| Laurales | Lamiaceae | *Vitex rotundifolia* L.f. | SS |
| Ranunculales | Lardizabalaceae | *Akebia chingshuiensis* T. Shimizu | TSd |
| Ranunculales | Lardizabalaceae | *Akebia longeracemosa* Matsum. | TSd |
| Ranunculales | Lardizabalaceae | *Stauntonia hexaphylla* (Thunb.) Dcene. | TSd |
| Ranunculales | Lardizabalaceae | *Stauntonia obovata* Hemsl. | TSd |
| Ranunculales | Lardizabalaceae | *Stauntonia purpurea* Y. C. Liu & F. Y. Lu* | TSd |
| Laurales | Lauraceae | *Cassytha filiformis* L. var. *duripraticola* Hatus. | TSd |
| Laurales | Lauraceae | *Cassytha filiformis* L. var. *filiformis* | TSd |
| Gentianales | Loganiaceae | *Gardneria multiflora* Makino | TSd |
| Gentianales | Loganiaceae | *Gardneria nutans* Siebold & Zucc. | TSd |
| Gentianales | Loganiaceae | *Strychnos cathayensis* Merr. | TMS |
| Malpighiales | Malpighiaceae | *Hiptage benghalensis* (L.) Kurz. | TSd |
| Malpighiales | Malpighiaceae | *Ryssopterys timoriensis* (DC.) Juss | TSd |
| Malpighiales | Malpighiaceae | *Tristellateia australasiae* A. Rich. | TSd |
| Malvales | Malvaceae | *Hibiscus surattensis* L. | SS |
| Myrtales | Melastomataceae | *Medinilla formosana* Hayata | SS |
| Myrtales | Melastomataceae | *Medinilla hayataina* H. Keng | SS |
| Ranunculales | Menispermaceae | *Cissampelos pareira* L. var. *hirsute* (DC.) Forman | TSd |
| Ranunculales | Menispermaceae | *Cocculus orbiculatus* (L.) DC. | TSd |
| Ranunculales | Menispermaceae | *Cyclea gracillima* Diels | TSd |
| Ranunculales | Menispermaceae | *Cyclea insularis* (Makino) Hatus. | TSd |
| Ranunculales | Menispermaceae | *Cyclea ochiaiana* (Yamam.) S. F. Huang & T. C. Huang* | TSd |
| Ranunculales | Menispermaceae | *Paratinospora dentata*(Diels) Wei Wang* | TSd |
| Ranunculales | Menispermaceae | *Pericampylus glaucus* (Lam.) Merr. | TSd |
| Ranunculales | Menispermaceae | *Sinomenium acutum* (Thunb.) Rehder & E. H. Wils. | TSd |
| Ranunculales | Menispermaceae | *Stephania cephalantha* Hayata | TSd |
| Ranunculales | Menispermaceae | *Stephania japonica* (Thunb.) Miers | TSd |
| Ranunculales | Menispermaceae | *Stephania longa* Lour. | TSd |
| Ranunculales | Menispermaceae | *Stephania merrillii* Diels | TSd |
| Ranunculales | Menispermaceae | *Stephania tetraandra* S. Moore | TSd |
| Ranunculales | Menispermaceae | *Tinospora crispa* (L.) J. D. Hook. & Thom. | TSd |
| Rosales | Moraceae | *Ficus aurantiaca* Griff. var. *parvifolia* (Corner) Corner | AR |
| Rosales | Moraceae | *Ficus pumila* L. var. *awkeotsang* (Makino) Corner | AR |
| Rosales | Moraceae | *Ficus pumila* L.var. *pumila* | AR |
| Rosales | Moraceae | *Ficus sarmentosa* Buch.-Ham. ex Sm. var. *henryi* (King ex D. Oliver) Corner | AR |
| Rosales | Moraceae | *Ficus sarmentosa* Buch.-Ham. ex Sm. var. *nipponica* (Franch. & Sav.) Corner | AR |
| Rosales | Moraceae | *Ficus trichocarpa* Blume var. *obtusa* (Hassk.) Corner | AR |
| Rosales | Moraceae | *Ficus vaccinioides* Hemsl. ex King* | AR |
| Rosales | Moraceae | *Maclura cochinchinensis* (Lour.) Corner | SS |
| Rosales | Moraceae | *Malaisia scandens* (Lour.) Planch. | TSd |
| Caryophyllales | Nyctaginaceae | *Bougainvillea spectabilis* Willdenow | SS |
| Caryophyllales | Nyctaginaceae | *Pisonia aculeata* L. | SS |
| Lamiales | Oleaceae | *Jasminum lanceolarium* Roxb. | TSd |
| Lamiales | Oleaceae | *Jasminum nervosum* Lour. | TSd |
| Lamiales | Oleaceae | *Jasminum sinense* Hemsl. | TSd |
| Lamiales | Oleaceae | *Jasminum urophyllum* Hemsl. | TSd |
| Santalales | Opiliaceae | *Cansjera rheedei* J. F. Gmelin | SS |
| Pandanales | Pandanaceae | *Freycinetia formosana* Hemsl. | AR |
| Malpighiales | Passifloraceae | *Passiflora biflora* Lam. | TPI |
| Malpighiales | Passifloraceae | *Passiflora edulis* Sims | TPI |
| Malpighiales | Passifloraceae | *Passiflora foetida* L. var. *tainaniana* Y.C. Liu & C.H. Ou | TPI |
| Malpighiales | Passifloraceae | *Passiflora laurifolia* L. | TPI |
| Malpighiales | Passifloraceae | *Passiflora ligularis* Juss. | TPI |
| Malpighiales | Passifloraceae | *Passiflora quadrangularis* L. | TPI |
| Malpighiales | Passifloraceae | *Passiflora suberosa* L. subsp. *litoralis* (Kunth) K.Port.-Utl. ex M.A.M.Azevedo, Baumbratz & Gonç.-Estev. | TPI |
| Malpighiales | Passifloraceae | *Passiflora vesicaria* L. | TPI |
| Malpighiales | Phyllanthaceae | *Phyllanthus reticulatus* Poiret | SS |
| Piperales | Piperaceae | *Piper arborescens* Roxb. | AR |
| Piperales | Piperaceae | *Piper betle* L. | AR |
| Piperales | Piperaceae | *Piper interruptum* Opiz | AR |
| Piperales | Piperaceae | *Piper kadsura* (Choisy) Ohwi | AR |
| Piperales | Piperaceae | *Piper kawakamii* Hayata | AR |
| Piperales | Piperaceae | *Piper* *lanyuense* K.N. Kung & Kun C. Chang* | AR |
| Piperales | Piperaceae | *Piper sarmentosum* Roxb. | SS |
| Piperales | Piperaceae | *Piper sintenense* Hatusima* | AR |
| Piperales | Piperaceae | *Piper taiwanense* T.T. Lin & S.Y. Lu* | AR |
| Caryophyllales | Polygonaceae | *Antigonon leptopus* Hook. & Arn. | TPI |
| Caryophyllales | Polygonaceae | *Persicaria chinensis* (L.) H.Gross | SS |
| Caryophyllales | Polygonaceae | *Persicaria perfoliata* (L.) H. Gross | SS |
| Caryophyllales | Polygonaceae | *Persicaria senticosa* (Meisn.) H. Gross ex Nakai | SS |
| Caryophyllales | Polygonaceae | *Reynoutria multiflorum* (Thunb.) Moldenke var. *hypoleuca* (Ohwi) S.S. Ying* | TSd,TSs |
| Ericales | Primulaceae | *Embelia laeta* (L.) Mez var. *papilligera* (Nakai) Walker | SS,TSd,TSs |
| Ericales | Primulaceae | *Embelia lenticellata* Hayata | TSd |
| Ericales | Primulaceae | *Embelia rudis* Hand.-Mazz. | TSs |
| Ranunculales | Ranunculaceae | *Clematis akoensis* Hayata* | TLp |
| Ranunculales | Ranunculaceae | *Clematis chinensis* Osbeck var. *chiensis* | TLp |
| Ranunculales | Ranunculaceae | *Clematis chinensis* Osbeck var. *tatushanensis* T.Y.A. Yang* | TLp |
| Ranunculales | Ranunculaceae | *Clematis crassifolia* Benth. | TLp |
| Ranunculales | Ranunculaceae | *Clematis formosana* Kuntz. * | TLp |
| Ranunculales | Ranunculaceae | *Clematis gouriana* Roxb. *ex* DC. subsp. *lishanensis* Yang & Huang * | TLp |
| Ranunculales | Ranunculaceae | *Clematis grata* Wall. | TLp |
| Ranunculales | Ranunculaceae | *Clematis henryi* Oliv. var. *henryi* | TLp |
| Ranunculales | Ranunculaceae | *Clematis henryi* var. *morii* (Hayata) T.Y.A. Yang & T.C. Huang* | TLp |
| Ranunculales | Ranunculaceae | *Clematis lasiandra* Maxim. | TLp |
| Ranunculales | Ranunculaceae | *Clematis leschenaultiana* DC. | TLp |
| Ranunculales | Ranunculaceae | *Clematis meyeniana* Walp. | TLp |
| Ranunculaceae | Ranunculaceae | *Clematis montana* Buch.-Ham. *ex* DC. | TLp |
| Ranunculaceae | Ranunculaceae | *Clematis parviloba Gard. ex Champ.* subsp*. bartlettii* (Yamamoto) Yang & Huang *** | TLp |
| Ranunculales | Ranunculaceae | *Clematis pseudootophora* M.Y. Fang | TLp |
| Ranunculales | Ranunculaceae | *Clematis tamurae* T. Y. A. Yang & T. C. Huang | TLp |
| Ranunculales | Ranunculaceae | *Clematis tashiroi* Maxim. var. *huangii* T.Y.A. Yang* | TLp |
| Ranunculales | Ranunculaceae | *Clematis tashiroi* Maxim. var. *tashiroi* | TLp |
| Ranunculales | Ranunculaceae | *Clematis terniflora* DC. var. *garanbiensis* (Hayata) M. C. Chang* | TLp |
| Ranunculales | Ranunculaceae | *Clematis uncinata* Champ. ex Benth. var. *okinawensis* (Ohwi) Ohwi | TLp |
| Ranunculales | Ranunculaceae | *Clematis uncinata* Champ. ex Benth.var. *uncinata* | TLp |
| Rosales | Rhamnaceae | *Berchemia arisanensis* Y. C. Liu & F. Y. Lu* | TSs |
| Rosales | Rhamnaceae | *Berchemia fenchifuensis* C. M. Wang & F. Y. Lu* | TSs |
| Rosales | Rhamnaceae | *Berchemia formosana* C. K. Schneid. | TSs |
| Rosales | Rhamnaceae | *Berchemia lineata* (L.) DC. | TSs |
| Rosales | Rhamnaceae | *Berchemia racemosa* Siebold & Zucc. var. *magna* Makino | TSs |
| Rosales | Rhamnaceae | *Rhamnus formosana* Matsum.* | SS |
| Rosales | Rhamnaceae | *Sageretia randaiensis* Hayata* | SS |
| Rosales | Rhamnaceae | *Sageretia thea* (Osbeck) M. C. Johnston | SS |
| Rosales | Rhamnaceae | *Ventilago elegans* Hemsl.* | PB |
| Rosales | Rhamnaceae | *Ventilago leiocarpa* Benth. | PB |
| Rosales | Rosaceae | *Rosa bracteata* Wendl. | SS |
| Rosales | Rosaceae | *Rosa cymosa* Tratt. | SS |
| Rosales | Rosaceae | *Rosa kwangtungensis* T.T. Yu & H.T. Tsai | SS |
| Rosales | Rosaceae | *Rosa laevigata* Michx. | SS |
| Rosales | Rosaceae | *Rosa luciae* Franch. & Rochebr. ex Crepinvar. var. *rosea* H. L. Li* | SS |
| Rosales | Rosaceae | *Rosa pricei* Hayata* | SS |
| Rosales | Rosaceae | *Rosa sambucina* Koidz. | SS |
| Rosales | Rosaceae | *Rosa transmorrisonensis* Hayata | SS |
| Rosales | Rosaceae | *Rubus* × *parvifraxinifolius* Hayata* | SS |
| Rosales | Rosaceae | *Rubus alceifolius* Poir. | SS |
| Rosales | Rosaceae | *Rubus amphidasys* Focke | SS |
| Rosales | Rosaceae | *Rubus buergeri* Miq. | SS |
| Rosales | Rosaceae | *Rubus cardotii* Koidz.* | SS |
| Rosales | Rosaceae | *Rubus corchorifolius* L. f. | SS |
| Rosales | Rosaceae | *Rubus croceacanthus* H. Lév. | SS |
| Rosales | Rosaceae | *Rubus flagelliflorus* Focke | SS |
| Rosales | Rosaceae | *Rubus formosensis* Kuntze | SS |
| Rosales | Rosaceae | *Rubus fraxinifolius* Poir. | SS |
| Rosales | Rosaceae | *Rubus howii* Merr. & Chun | SS |
| Rosales | Rosaceae | *Rubus incanus* Sasaki ex T.-S. Liu & T.-Y. Yang* | SS |
| Rosales | Rosaceae | *Rubus inopertus* (Focke ex Diels) Focke | SS |
| Rosales | Rosaceae | *Rubus lambertianus* Ser. var. *lambertianus* | SS |
| Rosales | Rosaceae | *Rubus lambertianus* Ser. var. *morii* (Hayata) S.-S. Ying* | SS |
| Rosales | Rosaceae | *Rubus linearifoliolus* Hayata | SS |
| Rosales | Rosaceae | *Rubus liui* Y.-P. Yang & S.-Y. Lu* | SS |
| Rosales | Rosaceae | *Rubus mesogaeus* Focke | SS |
| Rosales | Rosaceae | *Rubus nagasawanus* Koidz var. *arachnoideus* (Y.-C. Liu& F.-Y. Lu) S.-S. Ying* | SS |
| Rosales | Rosaceae | *Rubus nagasawanus* Koidz. var. *nagasawanus** | SS |
| Rosales | Rosaceae | *Rubus niveus* Thunb. | SS |
| Rosales | Rosaceae | *Rubus parviaraliifolius* Hayata* | SS |
| Rosales | Rosaceae | *Rubus parvifolius* L. | SS |
| Rosales | Rosaceae | *Rubus pectinellus* Maxim. | SS |
| Rosales | Rosaceae | *Rubus pentalobus* Hayata* | SS |
| Rosales | Rosaceae | *Rubus pungens* Cambess. var. *oldhamii* (Miq.) Maxim. | SS |
| Rosales | Rosaceae | *Rubus pyrifolius* Sm. | SS |
| Rosales | Rosaceae | *Rubus reflexus* Ker Gawl. var. *hui* (Diels ex Hu) F. P. Metcalf | SS |
| Rosales | Rosaceae | *Rubus rolfei* S. Vidal | SS |
| Rosales | Rosaceae | *Rubus rosifolius* Sm. | SS |
| Rosales | Rosaceae | *Rubus rufus* Focke | SS |
| Rosales | Rosaceae | *Rubus sumatranus* Miq. | SS |
| Rosales | Rosaceae | *Rubus swinhoei* Hance var. *kawakamii* (Hayata) S.-C. Liu* | SS |
| Rosales | Rosaceae | *Rubus swinhoei* Hance var. *swinhoei* | SS |
| Rosales | Rosaceae | *Rubus taitoensis* Hayata* | SS |
| Rosales | Rosaceae | *Rubus tephrodes* Hance var. *setosissimus*Hand.-Mazz. | SS |
| Rosales | Rosaceae | *Rubus trianthus* Focke | SS |
| Rosales | Rosaceae | *Rubus wallichianus* Wight & Arn. | SS |
| Gentianales | Rubiaceae | *Coptosapelta diffusa* (Champ. ex Benth.) Steenis | TSd |
| Gentianales | Rubiaceae | *Dimetia hedyotidea* (DC.) T. C. Hsu | TSd |
| Gentianales | Rubiaceae | *Gynochthodes parvifolia* (Bartl. ex DC.) Razafim. & B.Bremer | TSd |
| Gentianales | Rubiaceae | *Gynochthodes umbellata* (L.) Razafim. & B.Bremer | TSd |
| Gentianales | Rubiaceae | *Mussaenda formosanum* (Matsum.) T. Y. Aleck Yang & K. C. Huang* | TSs |
| Gentianales | Rubiaceae | *Mussaenda parviflora* Miq. | TSs |
| Gentianales | Rubiaceae | *Mussaenda pubescens* W. T. Aiton | TSs |
| Gentianales | Rubiaceae | *Mussaenda taihokuensis* Masam. | TSs |
| Gentianales | Rubiaceae | *Paederia cavaleriei* H. Lév. | TSs |
| Gentianales | Rubiaceae | *Paederia foetida* L. | TSs |
| Gentianales | Rubiaceae | *Psychotria serpens* L. | AR |
| Gentianales | Rubiaceae | *Randia sinensis* (Lour.) Roem. & Schult. | SS |
| Gentianales | Rubiaceae | *Rubia akane* Nakai var. *akane* | SS |
| Gentianales | Rubiaceae | *Rubia lanceolata* Hayata* | SS |
| Gentianales | Rubiaceae | *Rubia linii* C. Y. Chao* | SS |
| Gentianales | Rubiaceae | *Uncaria hirsuta* Havil. | HG |
| Gentianales | Rubiaceae | *Uncaria lanosa* Wall. var. *appendiculata* Ridsdale | HG |
| Gentianales | Rubiaceae | *Uncaria rhynchophylla* (Miq.) Miq. ex Havil. | HG |
| Sapindales | Rutaceae | *Toddalia asiatica* (L.) Lam. | SS |
| Sapindales | Rutaceae | *Zanthoxylum nitidum* (Roxb.) DC. | SS |
| Sapindales | Rutaceae | *Zanthoxylum scandens* Blume | SS |
| Proteales | Sabiaceae | *Sabia swinhoei* Hemsl. | TSd |
| Proteales | Sabiaceae | *Sabia transarisanensis* Hayata* | TSd |
| Sapindales | Sapindaceae | *Cardiospermum halicacabum* L. | TPI |
| Austrobaileyales | Schisandraceae | *Kadsura japonica* (L.) Dunal | TSs |
| Austrobaileyales | Schisandraceae | *Kadsura matsudae* Hayata | TSs |
| Austrobaileyales | Schisandraceae | *Kadsura oblongifolia* Merr. | TSs |
| Austrobaileyales | Schisandraceae | *Kadsura philippinensis* Elmer | TSs |
| Austrobaileyales | Schisandraceae | *Schisandra arisanensis* Hayata* | TSs |
| Liliales | Smilacaceae | *Smilax arisanensis* Hayata | TPD,SS |
| Liliales | Smilacaceae | *Smilax bracteata* C. Presl subsp. *bracteata* | TPD,SS |
| Liliales | Smilacaceae | *Smilax bracteata* C. Presl subsp. *verruculosa* (Merr.) T. Koyama | TPD,SS |
| Liliales | Smilacaceae | *Smilax china* L. | TPD,SS |
| Liliales | Smilacaceae | *Smilax corbularia* Kunch | TPD |
| Liliales | Smilacaceae | *Smilax discotis* Warb. subsp. *concolor* (Norton) T. Koyama* | TPD |
| Liliales | Smilacaceae | *Smilax elongato-umbellata* Hayata* | TPD, SS |
| Liliales | Smilacaceae | *Smilax glabra* Roxb. | TPD |
| Liliales | Smilacaceae | *Smilax horridiramula* Hayata* | TPD,SS |
| Liliales | Smilacaceae | *Smilax insularis* T.C. Hsu & S.W. Chung* | TPD |
| Liliales | Smilacaceae | *Smilax koyamae* T.C. Hsu & S.W. Chung* | TPD |
| Liliales | Smilacaceae | *Smilax lanceifolia* Roxb. | TPD |
| Liliales | Smilacaceae | *Smilax luei* T. Koyama* | TPD |
| Liliales | Smilacaceae | *Smilax nantoensis* T. Koyama* | TPD |
| Liliales | Smilacaceae | *Smilax nipponica* Miq. | TPD |
| Liliales | Smilacaceae | *Smilax ocreata* A. DC. | TPD,SS |
| Liliales | Smilacaceae | *Smilax plenipedunculata* Hayata var. *plenipedunculata** | TPD |
| Liliales | Smilacaceae | *Smilax plenipedunculata* Hayata var. *raishaensis* (Hayata) T.C. Hsu & S.W. Chung* | TPD |
| Liliales | Smilacaceae | *Smilax riparia* A. DC. | TPD |
| Liliales | Smilacaceae | *Smilax seisuiensis* (Hayata) T.C. Hsu & S.W. Chung | TPD |
| Liliales | Smilacaceae | *Smilax septemnervia* (F. T. Wang & T. Tang) T. C. Hsu & S. W. Chung | TPD |
| Liliales | Smilacaceae | *Smilax sieboldii* Miq. | TPD,SS |
| Liliales | Smilacaceae | *Smilax taipeiensis* T.C. Hsu & S.W. Chung* | TPD |
| Solanales | Solanaceae | *Solanum lyratum* Thunb. | TSs |
| Solanales | Solanaceae | *Solanum pittosporifolium* Hemsl. | TSs |
| Solanales | Solanaceae | *Solanum seaforthianum* Andrews | TLp |
| Crossosomatales. | Stachyuraceae | *Stachyurus himalaicus* Hook.f. & Thomson ex Benth. | SS |
| Pandanales | Stemonaceae | *Stemona tuberosa* Lour. | TSd |
| Rosales | Urticaceae | *Poikilospermum acuminata* (Trécul) Merr. | AR |
| Vitales | Vitaceae | *Ampelopsis delavayana* Planchon ex Franchet | TPI |
| Vitales | Vitaceae | *Ampelopsis glandulosa* (Wall.) Momiy. var. *glandulosa* | TPI |
| Vitales | Vitaceae | *Ampelopsis glandulosa* (Wall.) Momiy. var. *hancei* (Planch.) Momiy. | TPI |
| Vitales | Vitaceae | *Ampelopsis glandulosa* (Wall.) Momiy. var. *heterophylla* (Thunb.) Momiy. | TPI |
| Vitales | Vitaceae | *Ampelopsis japonica*(Thunb.) Makino | TPI |
| Vitales | Vitaceae | *Causonis corniculata* (Benth.) J.Wen & L.M.Lu | TPI |
| Vitales | Vitaceae | *Causonis japonica* (Thunb.) Raf. | TPI |
| Vitales | Vitaceae | *Causonis maritima* (Jackes) Jackes | TPI |
| Vitales | Vitaceae | *Causonis tenuifolia* (Wight & Arn.) G.Parmar & L.M.Lu | TPI |
| Vitales | Vitaceae | *Cissus assamica* (Laws.) Craib | TPI |
| Vitales | Vitaceae | *Cissus kerrii* Craib | TPI |
| Vitales | Vitaceae | *Cissus pteroclada* Hayata | TPI |
| Vitales | Vitaceae | Cissus repanda (Wight & Arn.) Vahl | TPI |
| Vitales | Vitaceae | *Cissus verticillata* (L.) Nicolson & C.E.Jarvis. | TPI |
| Vitales | Vitaceae | *Nekemias cantoniensis* (Hook. & Arn.) J.Wen & Z.L.Nie var. *cantoniensis* | TPI |
| Vitales | Vitaceae | *Nekemias cantoniensis* (Hook. & Arn.) J.Wen & Z.L.Nie var. *leecoides* (Maxim.) F.Y.Lu | TPI |
| Vitales | Vitaceae | *Parthenocissus tricuspidata* (Siebold & Zucc.) Planch | TPI,AR |
| Vitales | Vitaceae | *Pseducocayratia pengiana* Hsu & J.Wen* | TPI |
| Vitales | Vitaceae | *Tetrastigma formosanum* (Hemsley) Gagnepain* | TPI |
| Vitales | Vitaceae | *Tetrastigma hemsleyanum* Diels & Gilg | TPI |
| Vitales | Vitaceae | *Tetrastigma lanyuense* C. E. Chang* | TPI |
| Vitales | Vitaceae | *Tetrastigma obtectum* (Wallich ex M. A. Lawson) Planchon ex Franchet var. *glabrum* (H. Léveillé) Gagnepain | TPI,AR |
| Vitales | Vitaceae | *Tetrastigma obtectum* (Wallich ex M. A. Lawson) Planchon ex Franchet var. *obtectum* | TPI,AR |
| Vitales | Vitaceae | *Vitis amurenensis* Rupr. | TPI |
| Vitales | Vitaceae | *Vitis flexuosa* Thunberg | TPI |
| Vitales | Vitaceae | *Vitis heyneana* Roemer & Schultes subsp. *heyneana* | TPI |
| Vitales | Vitaceae | *Vitis heyneana* Roemer & Schultes subsp. *ficifolia* (Bunge)  C. L. Li, Chin | TPI |
| Vitales | Vitaceae | *Vitis sinocinerea* W. T. Wang | TPI |
| Vitales | Vitaceae | *Yua thomsonii* (M. A. Lawson) C. L. Li | TPI |

Note: the family arrangement according to Stevens (2001) Onwards. Angiosperm phylogeny website. Version 14, July 2017. *endemic,.TS: twining stem, TSd: twining stem in dextrorse, TSs: twining stem in sinistrorse, TMS: twining modified shoot, TTL; twining terminal leaflets, TLt: twining leaf tip, TPD: twining petiole duplication, TLp: twining leaf petioles, TPI: twining peduncles or inflorescence, PB: prehensile branch (also called TB: twining lateral branch), SS: simple scrambling, AR: adhesive roots, or adhesive pads, HG: hooks or grapnels, TT: twining tendrils (including TMS, TTL, TLt, TPD).
